# Intrinsic Resistance to EGFR-Tyrosine Kinase Inhibitors in *EGFR*-Mutant Non-Small Cell Lung Cancer: Differences and Similarities with Acquired Resistance

**DOI:** 10.3390/cancers11070923

**Published:** 2019-07-01

**Authors:** Eric Santoni-Rugiu, Linea C. Melchior, Edyta M. Urbanska, Jan N. Jakobsen, Karin de Stricker, Morten Grauslund, Jens B. Sørensen

**Affiliations:** 1 Department of Pathology, Rigshospitalet, Copenhagen University Hospital, DK-2100 Copenhagen, Denmark; 2 Department of Oncology, Rigshospitalet, Copenhagen University Hospital, DK-2100 Copenhagen, Denmark; 3 Department of Oncology and Palliative Units, Zealand University Hospital, DK-4700 Næstved, Denmark; 4 Department of Clinical Genetics and Pathology, Skåne University Hospital, SE-221 85 Lund, Sweden

**Keywords:** EGFR-mutated non-small cell lung cancer, EGFR-TKI, intrinsic resistance, resistance mechanisms

## Abstract

Activating mutations in the *epidermal growth factor* *receptor* gene occur as early cancer-driving clonal events in a subset of patients with non-small cell lung cancer (NSCLC) and result in increased sensitivity to EGFR-tyrosine-kinase-inhibitors (EGFR-TKIs). Despite very frequent and often prolonged clinical response to EGFR-TKIs, virtually all advanced *EGFR*-mutated (*EGFR*M+) NSCLCs inevitably acquire resistance mechanisms and progress at some point during treatment. Additionally, 20–30% of patients do not respond or respond for a very short time (<3 months) because of intrinsic resistance. While several mechanisms of acquired EGFR-TKI-resistance have been determined by analyzing tumor specimens obtained at disease progression, the factors causing intrinsic TKI-resistance are less understood. However, recent comprehensive molecular-pathological profiling of advanced *EGFR*M+ NSCLC at baseline has illustrated the co-existence of multiple genetic, phenotypic, and functional mechanisms that may contribute to tumor progression and cause intrinsic TKI-resistance. Several of these mechanisms have been further corroborated by preclinical experiments. Intrinsic resistance can be caused by mechanisms inherent in *EGFR* or by *EGFR*-independent processes, including genetic, phenotypic or functional tumor changes. This comprehensive review describes the identified mechanisms connected with intrinsic EGFR-TKI-resistance and differences and similarities with acquired resistance and among clinically implemented EGFR-TKIs of different generations. Additionally, the review highlights the need for extensive pre-treatment molecular profiling of advanced NSCLC for identifying inherently TKI-resistant cases and designing potential combinatorial targeted strategies to treat them.

## 1. Introduction

Uncontrolled activity of the transmembrane receptor tyrosine kinase (RTK) epidermal growth factor receptor (EGFR) can function as oncogenic driver and target for precision medicine intervention in lung cancer cells [1]. Once activated, EGFR undergoes auto-phosphorylation of tyrosine residues in its intracellular domain, recruits different adaptors and signal-transducers, and activates downstream signaling-pathways, such as the RAt Sarcoma small GTPase(RAS)-RAF proto-oncogene serine/threonine-protein kinase(RAF)-MAPK/ERK Kinase(MEK)-Mitogen-Activated Protein Kinase(MAPK), the Phosphatidyl-Inositol 3-Kinase(PI3K)-AKT serine/threonine kinase(AKT)-Phosphatase and TEnsin homolog(PTEN)-mammalian Target Of Rapamycin(mTOR), and the Signal Transducer and Activator of Transcription(STAT) pathways, thereby stimulating cellular proliferation, survival, protein synthesis, and migration as well as angiogenesis. Non-small cell lung cancer (NSCLC) in most cases is diagnosed in a locally advanced or disseminated stage and has poor prognosis. However, the advent of targeted therapy has provided, previously unmet, clinical benefit to subsets of patients with specific genetic cancer-drivers. Patients with *EGFR*-mutated (*EGFR*M+) NSCLC represent thus far the largest and most characterized of these NSCLC-subgroups. Activating *EGFR*-mutations occur in 10–35% of NSCLC cases, almost all of the lung adenocarcinoma (LAC) type, with significant ethnic variations (8–15% of LACs occur in Caucasians and 30–60% in East Asian populations) and a higher incidence among females, non-smokers, and patients younger than NSCLC patients with wild-type (wt) *EGFR* [1,2,3,4,5,6,7]. *EGFR-*mutations are extremely rare in pure pulmonary squamous cell carcinomas (SqCCs) and their occasional detection in this type of NSCLC has been ascribed by some authors to the misdiagnosis of cases that are adenosquamous carcinomas, or poorly differentiated LACs [8,9]. Tumor stage seems to affect the mutation rate too. A recent study by the Memorial Sloan Kettering Integrated Mutation Profiling of Actionable Cancer Targets (MSK-IMPACT) group showed an incidence of *EGFR*-mutations of 27% in a large cohort of multi-treated recurrent/metastatic LACs [10], as opposed to the frequency of 11% reported in The Cancer Genome Atlas (TCGA) cohort, which mainly consisted of non-metastatic, surgically removed LACS that had not received systemic treatment [3].

Exon 19-microdeletions (exon 19dels) or deletion-insertions (exon 19delins), most commonly occurring at the p.E746-A750 region and less frequently involving other positions between E746 and I759, and the point-substitution p.L858R (L858R) in exon 21, represent together nearly 90% of all *EGFR*-mutations in NSCLC [1,2]. These common mutations result in constitutive ligand-independent EGFR-TK activity and in increased affinity and sensitivity to EGFR-tyrosine-kinase-inhibitors (EGFR-TKIs) of first-generation (1G; gefitinib, erlotinib) and second-generation (2G; afatinib, dacomitinib) [4]. Exon 19dels and L858Rare almost exclusively early clonal events (founder mutations) involved in tumor initiation during the evolution of LAC, thus explaining the significant and uniform responses that are often observed across multiple cancer sites when these mutations are targeted by TKIs [7,11,12]. The 1G EGFR-TKIs reversibly bind to the ATP-binding site of the intracellular TK-domain of EGFR, thereby impeding the autophosphorylation of EGFR and the activation of the downstream signaling-pathways, whereas 2G TKIs irreversibly bind and inhibit not only the TK-domain of EGFR, but also of other ERBB-family members, such as ERBB2 and ERBB4. Given these properties, 1G and 2G EGFR-TKIs for several years have represented the standard of care (SOC) first-line treatment for advanced *EGFR*M+NSCLC, with the choice of first-line between 1G and 2G mostly linked to different toxicity profiles and mutation types, as afatinib is associated with more frequent side effects and is more effective in NSCLC cases harboring exon 19dels and uncommon *EGFR*-mutations than in patients with L858R [13,14]. However, the initial response is transient and virtually all *EGFR*M+ NSCLCs inevitably become resistant to first-line EGFR-TKIs, with a median progression-free survival (PFS) of 9–13 months [15,16]. Approximately 60% of cases of acquired resistance to 1G TKIs are due to the secondary p.T790M (T790M) *EGFR*-mutation in exon 20, which does activate EGFR, but possesses also an increased affinity for ATP that competitively hampers the binding of reversible EGFR-TKIs to the EGFR ATP-binding pocket [17,18]. The frequency of T790M in cases progressing during treatment with the 2G TKI afatinib seems to be even higher, reportedly more than 73% [19].

Thus, the third-generation (3G), more central nervous system (CNS)-penetrant, irreversible EGFR-TKI, osimertinib, which selectively inhibits both EGFR-TKI-sensitizing mutations and T790M without binding wild-type (wt) EGFR, is approved worldwide as the SOC for second-line therapy of advanced T790M-positive NSCLC, given its superior efficacy over platinum-pemetrexed therapy in this setting, including in patients with CNS metastases [20,21]. In terms of the overall survival (OS) rate, more mature clinical trial data for osimertinib second-line (129 patients) or third- or later-line (282 patients) in pretreated T790M-mutant patients were recently reported, showing a median OS of 26.8 months and a 12-month, 24-month, and 36-month survival rate of 80%, 55%, and 37%, respectively, further supporting the choice of this drug in these patients [22]. Outside clinical trials, a recent retrospective multicentric study of T790M-positive patients confirmed the efficacy of second-/third-line osimertinib in a real-world setting, both in patients with and without cerebral metastases [23].

Importantly, osimertinib (at a dose of 80 mg once daily), in a recent comparison (FLAURA trial) with SOC 1G TKIs erlotinib and gefitinib in first-line management of treatment-naïve patients with advanced *EGFR*M+ NSCLC, exhibited superior efficacy (median PFS of 18.9 months vs. 10.2 months; hazard ratio (HR) 0.46; *p* < 0.001; median duration of response 17.2 months vs. 8.5 months), similar response rate (RR; 80% for osimertinib vs. 76% for SOC TKIs), and reduced frequency of serious adverse events (34% vs. 45%) [24]. Another study testing osimertinib as first-line in treatment-naïve patients with advanced *EGFR*M+ NSCLC showed a comparably robust RR (67% for patients receiving 80 mg/day, 87% for those receiving 160 mg/day) and protracted median PFS (22.1 months in the 80 mg group, 19.3 months in the 160 mg group) [25]. Furthermore, in patients with untreated *EGFR*M+ advanced NSCLC from the phase III FLAURA study, osimertinib, in keeping with its higher CNS penetrance, demonstrated superior CNS efficacy and reduced risk of CNS progression when compared with SOC first-line EGFR-TKIs [26]. Even if more mature data on osimertinib’s OS rate derived from the FLAURA trial are awaited, and despite some concerns related to its cost-effectiveness [27,28], osimertinib holds great promise as first-line treatment for patients with advanced *EGFR*M+ NSCLC. However, most T790-positive cases treated with this drug as second line become resistant within 9–13 months through different mechanisms that have been identified in tissue samples and plasma circulating free (cf)DNA [25,29,30]. The former mechanisms include most frequently tertiary *EGFR*-mutations (especially C797S, but also rarer mutants at codon L718/G719, G796/C797, L792, L798, and others) and more seldom *EGFR*-amplification or the reduction/disappearance of the T790M-mutation due to the emergence of “target-less” T790-negative clones [29,30,31,32,33].

The EGFR-independent mechanisms of acquired resistance resemble those underlying progression upon treatment with 1G/2G TKIs, i.e., the activation of by-pass pathways via the amplification (*ERBB2*/*HER2, MET, FGFR1, KRAS*) or fusion (*RET, ALK, FGFR3, NTRK1*) of alternative *RTK* genes as well as activating mutations or fusions of members of the downstream RAS-RAF-MEK-MAPK and PI3K/AKT/PTEN/mTOR pathways [25,29,30,32,34,35,36,37,38]. Interestingly, in some cases characterized by particularly rapid progression (including cases fulfilling the temporal definition of intrinsic resistance in Section 1.1) and poor survival on osimertinib, the appearance of *RTK-* or *BRAF*-fusions or *KRAS-*mutations coincided with the loss of the T790M mutation and preservation of the original activating *EGFR*-mutation [32,36,37]. This suggests either that osimertinib has eliminated the T790M-positive clones or that the cancer cells themselves have lost this osimertinib-target, thereby switching from T790M as acquired driver to another acquired driver such as RTK- or BRAF-fusion proteins. Additional mechanisms of acquired resistance shared by TKIs of all three generations are the phenotypic transformation to small-cell lung cancer (SCLC), the epithelial-mesenchymal transition (EMT), and the conversion to SqCC [29] (see Section 2.3.1, Section 2.3.2 and Section 2.3.3). Besides establishing the aspects of acquired TKI-resistance, recent years have also witnessed increasing focus on intrinsic resistance to EGFR-TKIs.

### 1.1. Intrinsic (Primary, Inherent) TKI-Resistance

Although most of the patients with advanced *EGFR*M+ NSCLC achieve objective response (OR) to TKIs, the extent and duration of responses are variable, and 20–30% of patients do not respond or respond for a very short time (typically <3 months) because of intrinsic resistance caused by de novo mechanisms believed to exist before treatment [15,39]. Thus, in intrinsic/primary resistance the inefficacy of TKIs is immediately or very rapidly discernable, while in acquired/secondary resistance, disease progression occurs after an objective and sometimes prolonged clinical benefit from TKI-treatment. This benefit has been defined as either radiologically documented complete or partial response (CR, PR) or durable (≥6 months) stable disease (SD; defined by response evaluation criteria in solid tumors (RECIST) or World Health Organization (WHO) criteria) after TKI initiation and uninterrupted exposure without receiving additional systemic therapy after TKI discontinuation [15,16]. While the differentiation of intrinsic from acquired resistance is based on temporal and objectively measurable criteria, it is likely that what we call “acquired resistance” may combine the expansion of original clones pre-existing prior to treatment (as in the “intrinsic resistance”) and new resistance mechanisms developed as a form of gradual adaptive response of cancer cells to the treatment. This explains why a certain number of mechanisms appear to be shared by the two types of resistance.

While several mechanisms of acquired EGFR-TKI resistance have been uncovered by analyzing tumor specimens obtained at disease progression [17,18,29], the factors influencing the initial response and causing primary resistance to TKIs have been less studied. However, comprehensive genomic profiling of tumor specimens by high-throughput next generation sequencing (NGS) analyses performed during the last decade has enabled the identification of the most frequent recurrent driver alterations, such as single nucleotide variants (SNVs)/point mutations, gene insertions and deletions (indels), copy number variations (CNVs), and overexpressed oncogenes that mediate the pathogenesis and progression of NSCLC, and that represent potential therapeutic targets [3,8,9,10,40,41]. In particular, associating the results obtained by whole genome/exome sequencing or by more focused hotspot mutation analysis using targeted NGS of selected gene panels with the response to EGFR-TKIs has elucidated in recent years the mechanisms of intrinsic resistance to these drugs.

The first large-scale genome sequencing studies on NSCLC were primarily based on resected early-stage tumors not treated with TKIs, thus they supported the predominant view of one single, usually “mutually exclusive”, oncogenic driver, like the mutated *EGFR* [3,8]. However, the following genomic analyses focusing on large cohorts of patients with advanced *EGFR*M+ NSCLC have challenged this view and shown that other important genetic alterations regulating multiple signaling pathways are commonly co-occurring and function as co-drivers contributing to tumor progression and drug-resistance, both in the intrinsic and acquired resistance settings [10,12]. In this review, we present the recurrent themes concerning intrinsic TKI-resistance that have emerged from these clinical studies, including significant similarities and differences between primary and secondary resistance. Furthermore, we discuss preclinical investigations in NSCLC cell lines and animal models that have elucidated or further corroborated specific mechanisms of inherent TKI-resistance.

## 2. Clinical and Preclinical Studies Shedding Light on Intrinsic Resistance to EGFR-TKIs

Although most of the activating *EGFR*-mutations occurring in NSCLC before treatment have for a long time been considered mutually exclusive with other recurrent cancer-driver alterations, more recent sensitive molecular analyses have shown the concomitant occurrence of other driver-mutations in a significant percentage of untreated *EGFR*M+ LACs [3,10,12,42,43,44,45,46,47]. A large French database study including 17664 lung cancer patients lately detected 2–3 concurrent driver-mutations in almost 1% of these cases before treatment [44]. Moreover, comparative genomic analysis of cfDNA from 1122 *EGFR*M+ and 1008 *EGFR*-wt patients with stage III/IV NSCLC illustrated the extensive co-occurrence of other crucial somatic genetic alterations in the *EGFR*M+ population before TKI-therapy [12]. Remarkably, this study revealed additional variants of functional significance in the cfDNA obtained from 93% of the *EGFR*M+ cases, with a mean of 2.58 ± 1.7 (S.E.M) genetic alterations beyond *EGFR* (out of 68 NGS-profiled genes) and a range of identified alterations of 1–13, when including *EGFR* [12]. Only 10% of the identified co-mutations were categorized as probable passenger events, while 90% of them were predicted to have a functional impact and act as co-drivers by affecting several genes down-stream *EGFR*, such as *MET, PIK3CA, BRAF, MYC, CDK6*, *AR, TP53, CTNNB1* and others. An enrichment of co-alterations in several genes potentially activating the Wnt/β-catenin pathway, hormonal signaling, and cell cycle was observed in the *EGFR*M+ cases as compared to those with *EGFR*-wt, suggesting the pathogenetic role of these genetic co-aberrations in advanced *EGFR*M+ NSCLC [12]. By longitudinal investigation of cfDNA samples obtained from patients who were EGFR-TKI-naïve or had progressed on first-/second-line EGFR-TKI treatment, the same authors described that although the number of detectable somatic genetic alterations increased with each line of therapy, co-alterations of certain driver-genes where already identifiable before TKI-start [12]. Furthermore, the mean number of functional genetic co-alterations detectable in cfDNA was lower in patients who responded to a subsequent EGFR-TKI (of any generation) compared to non-responders. Finally, co-alterations in *MET,* other genes of the MAPK, PI3K, and Wnt/β-catenin pathways or cell cycle genes were associated with poor response to EGFR-TKIs [12]. Jointly, these data imply that coexisting mutations in *EGFR* itself or in other cancer-drivers at baseline may potentially impair the efficacy of EGFR-TKIs and explain why some TKI-treated NSCLCs are intrinsically resistant [18]. This, in turn, means that we should expect an increased investigational and medical burden for NSCLC patients and economic burden for health systems, as additional therapies or drug combinations need to be implemented for tackling the problem of TKI-resistance. It also suggests that the current routine testing of *EGFR*, *ALK*, and *ROS1* performed on tumor tissue or plasma samples for selecting NSCLC patients treatable with first-line targeted therapy is actually not enough to predict the response to the approved TKIs.

The increasing availability of size-variable NGS panels can provide relevant information for both SOC predictive biomarkers and investigational treatment options based on the analysis of potentially actionable genetic events [10,48,49,50]. We recently addressed this topic too by evaluating the frequency of an extended panel of cancer-relevant mutations that could have possibly affected the initial response to erlotinib in a consecutive series of *EGFR*M+/*ALK*-negative/*ROS1*-negative advanced NSCLCs [51]. In this cohort, the initial *EGFR*-mutation status had been tested by the commercially available real-time/quantitative polymerase chain reaction (PCR)-based Cobas^®^ EGFR Mutation assay v2 (Roche Molecular Diagnostics), which is Food and Drug Administration (FDA)- and European Medicines Agency (EMA)-approved as a companion diagnostic test for erlotinib, gefitinib and osimertinib in tissue and liquid biopsy samples and can detect 42 known *EGFR*-mutations. The retrospective analysis of possible relevant co-alterations using targeted NGS, fluorescence in-situ hybridization (FISH), and immunohistochemistry (IHC) indeed indicated that concomitant occurrence of other mutations in *EGFR* itself or other genes may have an impact on the response to erlotinib [51]. Similarly, a retrospective analysis of cfDNA from a Chinese cohort of *EGFR*M+ NSCLC cases before treatment with 1G EGFR-TKIs detected co-existing mutations in *EGFR* or other cancer-relevant genes in 22% and 55% of patients, respectively, and showed that these co-alterations correlated with poor OR and OS after implementing these drugs [52]. Another recent retrospective study confirmed that a significant fraction of *EGFR*M+ NSCLCs harbors co-mutations in other genes, which can negatively affect the response to EGFR-TKIs [53].

**Figure 1:**
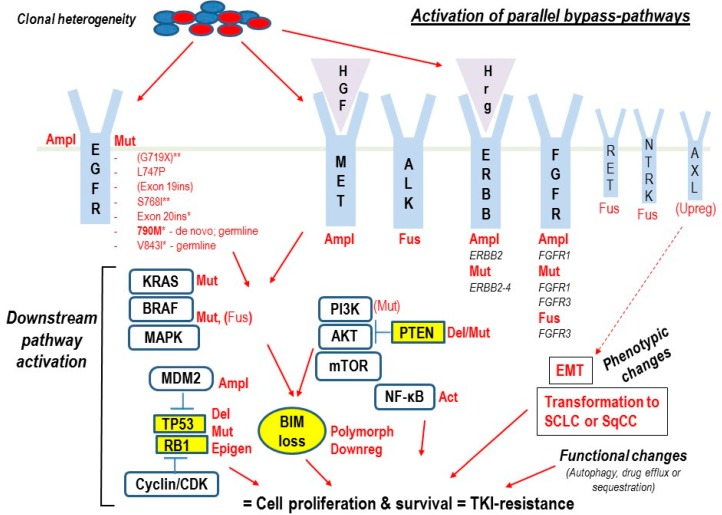
Summary of main molecular mechanisms of intrinsic resistance to EGFR-tyrosine-kinase-inhibitors (EGFR-TKIs) in *EGFR*-mutated (*EGFR*M+) non-small cell lung cancer (NSCLC). Given the clonal genetic heterogeneity of NSCLC, innate genetic alterations capable of impairing the response and causing intrinsic resistance to TKIs may be present in pre-existing clones before treatment (de novo alterations) or may be very rapidly induced in surviving cancer cells as immediate adaptive response or tolerance to the targeted therapy. If the relative allelic frequency of one or several (polyclonal resistance) of these pre-existing/immediately induced alterations is sufficient to very rapidly counteract the effect of TKIs (conventionally within the first 3 months after TKI-treatment initiation), tumor cells will continue to proliferate and survive, and intrinsic TKI-resistance will ensue. The EGFR-dependent resistance mechanisms are represented by amplification (**Ampl**) and/or specific somatic or germline mutations (**Mut**) of the *EGFR*-gene. Some of these mutations cause resistance to EGFR-TKIs of all three generations, while others are sensitive to 2G or 3G TKIs, as indicated (* = EGFR-mutants resistant to 1G/2G EGFR-TKIs, but sensitive to 3G TKIs, the most common being **T790M** indicated in bold; ** = EGFR-mutants resistant to 1G EGFR-TKIs, but sensitive to afatinib). Among the uncommon *EGFR*-mutations G719X and insertions in exon 19 (Exon 19ins) are indicated in brackets, because despite being less sensitive than common EGFR-mutants, they may show some response to 1G TKIs. Instead, most of the EGFR-independent resistance mechanisms are shared by EGFR-TKIs of all three generations and include the activation of by-pass pathways via amplification (**Ampl**), mutation (**Mut**) or fusion (**Fus**) of alternative parallel receptor tyrosine kinase- (RTK)-genes such as *Mesenchymal-Epithelial Transition* (*MET), Anaplastic Lymphoma Kinase (ALK)*, non-EGFR *Erythroblastic Oncogene B*(*ERBB)*-family-members, *Fibroblast Growth Factor Receptor* genes (*FGFR*s) (written in bold), and possibly *REarranged during Transfection* (*RET*) and *Neurotrophic Tyrosine Receptor Kinase (NTRK)* (not in bold). Activation of parallel RTKs can also be induced by overexpression of hepatocyte growth factor (HGF) that binds the MET-receptor or Heregulin (Hrg) that binds ERBB2. Alternative downstream by-pass mechanisms of resistance are represented by mutations, fusions, or deletion (**Del**) of members of the RAS-RAF-MEK-MAPK and PI3K-AKT-PTEN-mTOR pathways or inactivation of *TP53* and/or *retinoblastoma 1 (RB1)* tumor-suppressor genes via mutation/deletion/epigenetic mechanism (**Epigen**) or indirectly by gene-amplification of the p53-inhibitor Mouse Double Minute 2 homolog (MDM2) and mutation/amplification of genes encoding cyclins and cyclin-dependent kinases (CDKs). Additional by-pass mechanisms are activation (**Act**) of the NF-κB transcription factor by different pathways or impairment of TKI-induced apoptosis by loss of the pro-apoptotic *BIM*-gene expression due to genetic polymorphism (**Polym**) or transcriptional downregulation (**Downreg**). Putative mechanisms of intrinsic (and acquired) resistance to all three generations’ TKIs that await further clinical validation are phenotypic changes, such as epithelial-mesenchymal transition (EMT), transformation to small-cell lung cancer (SCLC) or squamous cell carcinomas (SqCC), and potential functional changes reducing TKI efficacy, like rapidly increased autophagic activity, drug-efflux or intracellular drug-sequestration in cancer cells. Some evidence for NSCLC cases with pre-existing, inherently TKI-resistant cells due to upregulation (Upreg; in brackets) of the EMT-inducing RTK AXL has also been provided. RTKs are in light blue, intracellular downstream oncoproteins in white boxes, tumor suppressors in yellow symbols.

In the following sections, we will discuss co-mutations and other factors that may affect the response to EGFR-TKIs and thereby represent inherent mechanisms of resistance to these drugs in NSCLC patients. Figure 1 summarizes the main mechanisms causing intrinsic resistance to EGFR-TKIs in NSCLC that have emerged from the recent preclinical and clinical studies detailed in the following sections. For *EGFR*-mutations and co-mutations involved in intrinsic TKI-resistance see also Table 1.

### 2.1. Impact of EGFR-Mutations or -Co-Mutations on Response to EGFR-TKIs

Published data clearly indicate that both the type and number of *EGFR*-mutations can impact the responsiveness to EGFR-TKIs of NSCLC patients. Given the higher incidence of single or combined *EGFR*-mutations in East-Asian NSCLC populations, most of the findings and interpretations on the effect of *EGFR* co-mutations (called complex mutations by some authors) on TKI-treatment come from studies in East-Asian cohorts. Moreover, a plethora of rare *EGFR*-mutations occurring alone or in combination with more common mutants have been occasionally observed in connection with disease progression after 1G EGFR-TKIs, both in the primary and acquired resistance setting [54]. A recognized general notion is that TKI-treated NSCLC patients with complex *EGFR*-mutations (≥2 different co-existing *EGFR*-mutations) show inferior RR and shorter PFS than patients with single *EGFR*-mutations, unless the combined mutations are the common exon19dels and L858R [55,56]. In this respect, we and others have identified by NGS analysis cases of advanced NSCLC with co-mutations in the *EGFR* gene differently affecting the treatment outcome, including cases showing no OR to erlotinib and co-existence at baseline of the L858R and the intrinsic erlotinib-resistant T790M *EGFR*-mutations [51]. *EGFR*-mutations that are insensitive to current TKIs represent a mechanism of “pharmacological” intrinsic resistance due to conformational changes in the drug-binding site of EGFR rather than to the biological signaling properties of the mutant EGFR-protein.

As for acquired resistance to 1G/2G EGFR-TKIs, T790M is the de novo *EGFR*-mutation most commonly associated with intrinsic resistance, whereas alternative secondary resistant *EGFR*-substitutions, such as L747S, D761Y, and T854A are much more rarely involved [57,58]. Several studies using conventional mutation analysis including Sanger sequencing, allele-specific PCR techniques, and NGS, occasionally (<1% incidence) detected the de novo T790M mutation at low allele frequency (AF) either alone or as a minor clone within treatment-naive specimens (biopsies or cfDNA) containing classic sensitizing *EGFR*-mutations [54,57,59]. The frequency of de novo T790M was 4.2% among 437 assessable patients in the randomized pan-Asian phase III IPASS trial [2], while it accounted for 2% (16/800) of all identified *EGFR*-mutant cases in a large Chinese cohort of 1903 resected NSCLCs [60] and was estimated to be <3% in Caucasians using Sanger sequencing [1]. Recently, two Chinese and one Italian NGS-based studies identified a de novo T790M co-mutation in 5.8%, 14%, and 6.8% of TKI-naïve patients concomitantly carrying sensitizing *EGFR*-mutations, respectively [52,53,61]. These data not only indicate that combined mutations may impact the occurrence of specific resistant *EGFR*-mutations such as T790M, but also that the higher sensitivity of NGS panels compared to previous DNA-sequencig methods may increase the frequency of T790M co-mutation.

As opposed to cases with T790M acquired during TKI-treatment, de novo T790M mutations more frequently coexist with L858R than with *EGFR* exon 19dels [51,54,61,62]. Importantly, meta-analysis showed that the identification of this association required sensitive methods (with detection limit of <5%), such as NGS, locked nucleic acid PCR or quantitative PCR [62]. In this regard, standard PCR followed by a modified colony hybridization technique with analytical sensitivity as low as 0.01% revealed the co-occurrence of de novo T790M at very low AF in 35–79% of TKI-naïve NSCLCs with sensitizing *EGFR*-mutations [63,64]. Although their clinical significance remains to be better determined, these findings do confirm that a substantial subgroup of patients with *EGFR*M+ NSCLC harbor some tumor cells with T790M co-mutation already before EGFR-TKI treatment. They also suggest that routine *EGFR*-mutation analysis at baseline should be performed with methods capable of detecting low-frequency co-mutations that could potentially impact response to TKI-treatment. Accordingly, early in vitro observations indicated that the co-presence of T790M may increase the oncogenic activity of common *EGFR*-mutants, such as exon 19dels and L858R [65], which may explain the possible occurrence of this mutant before TKI-treatment and its drug-induced selection as drug-resistant mutation during therapy.

The de novo and acquired T790M also seem to differ significantly in terms of the average relative allele frequency (RAF = AF of T790M/AF of sensitizing *EGFR*-mutation). Indeed, the RAF of T790M was reported higher in the de novo group (86.1% vs. 22.3%, *p*  <  0.0001) [61]. Consequently, the only patient achieving partial response (PR) among the 10 patients with de novo T790M that Tian et al. treated with erlotinib was the one with the lowest T790M RAF (19.7%), while the other nine patients with an average T790M RAF of 85.9% did not display OR. Notably, in the de novo group, the cases with the highest T790M RAF also harbored *EGFR* gene-amplification, possibly making them further TKI-resistant [61], as selective amplification of the T790M-containing allele represents a combination of two resistance mechanisms within the *EGFR*-gene [29,57]. This combination mechanistically resembles the *anaplastic lymphoma kinase* (*ALK)*-fusion gene-amplification detectable in certain *ALK*-positive patients becoming resistant to ALK-TKIs [57].

Collectively, the data infer that, as for any TKI-resistant mutation, a certain number (clone) of T790M-positive cells may co-exist with sensitizing *EGFR*-mutations in the heterogenous tumor tissue before treatment with 1G/2G TKIs and hamper the efficacy of these drugs [11,63,66]. If the RAF of T790M in this subclone is enough to immediately/very rapidly oppose the effect of TKI-treatment, it may result in intrinsic resistance, lack of OR, and poor outcome in patients receiving 1G/2G TKIs (Figure 1). If, instead, the RAF of T790M in the tumor tissue is too low to immediately counteract the TKI effect (in which case it will often be undetectable with routine analyses), the initial small population of T790M-positive cancer cells may be gradually expanded over time under the selective pressure generated by the TKI-treatment itself, until it can effectively counter the therapeutic effect of TKIs and cause secondary resistance and tumor progression after an initial OR or SD [66]. In this respect, a recent retrospective analysis of a relatively small phase II study of *EGFR*M+ NSCLC patients receiving afatinib plus bevacizumab combination therapy after becoming unresponsive to gefitinib suggested that this combined treatment could induce a more effective clonal selection of pre-existing T790M-positive cancer cells in heterogeneous tumors than gefitinib [67]. Therefore, according to the authors, this combined treatment could be exploited to provoke the conversion of T790M-negative patients into T790M-positive ones in order to allocate them to more effective treatment with osimertinib [67]. Although appealing as a potential therapeutic strategy, these notions await more validation in larger cohorts.

Likewise, the T790M RAF might be considered as predictive biomarker for treatment response to 1G and 2G EGFR-TKIs [61], but further research is needed to validate the possible clinical applicability and usefulness of this approach. For the time being, the simple detection of contemporaneous de novo T790M and sensitizing *EGFR*-mutations in NSCLC samples at baseline should be considered as an indication for employing osimertinib as first-line treatment in these cases. It is worthwhile underlining that a single gene alteration such as T790M may not necessarily be sufficient to cause EGFR-TKI resistance [12]. Large-scale genomic analysis of cfDNA from patients with advanced *EGFR*M+ NSCLC showed that specific genetic co-alterations in other cancer drivers (*CDK6, CCNE1, CTNNB1, AR, MYC, BRCA1*) may co-occur with T790M in advanced NSCLC, suggesting a collaborative functional role for these co-altered genes in driving EGFR-TKI resistance together with the T790M mutant [12]. This is consistent with the concept of polyclonal TKI-resistance [57] and with cases of *EGFR*M+ NSCLC displaying different mechanisms of TKI-resistance in separate metastatic sites [68].

Given the frequent clonal heterogeneity of NSCLCs, the lack of T790M in a single tumor biopsy at baseline cannot exclude a priori the occurrence of few tumor cells with de novo T790M intrinsically resistant to first-line TKIs. In fact, there is now compelling evidence for using cfDNA isolated from plasma and genotyped by PCR or NGS techniques as a valid tool for non-invasive assessment of the possible occurrence of T790M and other TKI-unresponsive mutations at baseline or acquired during treatment in patients, whose tumors are heterogeneous or inaccessible by tissue biopsy [12,48,52,69,70,71]. Liquid biopsies and tissue re-biopsies have shown high concordance for mutation-detection and for predicting the response to EGFR-TKIs of all three generations, supporting the applicability of cfDNA as tool to monitor the response to TKIs and to identify resistance-drivers [32,48,71,72]. Furthermore, it has been documented that the appearance and increase of resistance-mutations in cfDNA of TKI-treated patients with advanced *EGFR*M+ NSCLC is detectable several days (from 15 to 344 days) prior to radiological evidence of progression [73]. However, false negative results due to suboptimal sensitivity or non-shedding tumor clones may represent a limiting factor in certain cases, thus analysis of tissue biopsies, when feasible, remains the SOC and is recommendable when no resistance-causing mutations are identified in cfDNA at progression. In this regard, Ramalingam and co-workers were not able to clarify the mechanisms of resistance to first-line osimertinib in 50% of their patients that had been monitored using post-progression plasma samples (*n* = 38), because of lack of detectable circulating tumor DNA in these liquid biopsies [25]. Also relevant for detecting resistance-mutations and overcoming the problem of mutational tumor heterogeneity and missed mutations by bulk NGS, is the rapid development of high-throughput single-cell DNA sequencing and gene-expression analysis for assessing clonal evolution in tumors [74].

Regarding less common *EGFR*-mutations, the relative scarcity of clinical cases analyzed has precluded for quite some time the possibility of drawing firm conclusions on their response to different EGFR-TKIs in NSCLC patients and their possible role in intrinsic resistance. Yet, there is now accumulating evidence confirming that also some of the uncommon *EGFR*-mutations can negatively affect the response to TKIs. Focusing on the most relevant of these mutations, we and others have reported outcomes in erlotinib- or gefitinib-treated NSCLC cases carrying at baseline the exon 18 G719X (G719C, G719S, G719A or G719D), exon 20 S768I, or exon 21 L861Q *EGFR*-mutations, which are present in 1–8% of *EGFR*M+ NSCLCs and often occur simultaneously as complex mutants (G719X+S768I/L861Q) [1,51,75,76,77,78,79,80]. Together with even more uncommon single or complex (≥2 different co-existing) mutations in exon 18, 20 or 21, the G719X, S768I, and L861Q mutants, although structurally considered TKI-sensitizing [76,81], have shown in several case reports and retrospective case series treated with erlotinib or gefitinib significantly lower RR, shorter PFS, and worse OS compared to exon 19dels or L858R [54,56,75,77,78,79,80,81,82,83,84,85,86,87]. The frequency of these different uncommon mutations and the reported associated values for RR and survival vary among different reports, which is likely related to the retrospective character of these studies and the heterogenous cohorts analyzed. Interestingly, some studies found a significant association between these uncommon *EGFR*-mutations and smoking habits as opposed to the common exon 19dels and L858R that are much more frequent among non-smokers [80,85]. Also, some data indicate that these uncommon *EGFR*-mutants, although being often combined with each other in the same tumor, are rarely associated with mutations in other oncogenic drivers at baseline. This suggests that they may be sufficient for promoting tumor growth and for causing a sort of “pharmacological”, and not biological, intrinsic resistance to TKI-treatment (related to the conformation of the drug-binding pocket in these mutants), without the need for additional mechanisms [80,81].

A recent “real-world” study evaluating the efficacy and outcomes of treatment with 1G EGFR-TKIs vs. platinum-based chemotherapy in patients with advanced LAC harboring uncommon mutations alone or in combination [88] showed no significant difference in RR between the two groups (33% for EGFR-TKIs vs. 27% for chemotherapy, *p* = 0.5), which in both cases is far less than the RR in patients with common *EGFR*-mutations [2]. Interestingly, the PFS was 7.2 months among patients with uncommon mutations treated with 1G EGFR-TKIs compared to 4.9 months in the chemotherapy group (*p* = 0.00088), while the median OS was significantly worse in patients receiving TKIs than in those managed with chemotherapy (14.3 vs. 20.7 months, *p* = 0.0336). Thus, the study by Li et al. [88] confirms the reduced sensitivity to 1G EGFR-TKIs of the uncommon *EGFR*-mutants and suggests that longer OS may be achieved in these patients by adding chemotherapy to their management.

However, most patients with G719X, S768I or L861Q, alone or in combination with other mutations are significantly more responsive to afatinib (higher RR, longer PFS and OS than 1G EGFR-TKIs), whereas this drug is not effective in cases with de-novo T790M alone/combined with other mutations or with exon 20-insertions (exon 20ins) [81,89,90]. The combined post-hoc analysis of LUX-Lung 2, LUX-Lung 3, and LUX-Lung 6 indicated that the RR to afatinib was higher for patients with G719X (77.8%) than for those with L861Q (56.3%) [90]. In any case, the current indication for afatinib includes NSCLC-patients with exon 19dels or with the uncommon G719X, S768I or L861Q *EGFR*-substitutions, though patients with these mutations, over time, can become resistant to afatinib by acquiring a secondary T790M mutation or more rarely other substitutions in exon 20 [89,91]. Intriguingly, preclinical data suggest that G719X, S768I and L861Q are more sensitive to afatinib than to erlotinib or osimertinib [89,92] and that osimertinib has limited efficacy on NSCLC cells harboring these mutations, irrespective of the co-presence of T790M mutation [92]. Furthermore, among the seven NSCLC patients with G719X mutations included in the AURA trial for second-line osimertinib, only one (RR = 14%) showed partial response (PR), three (43%) had SD, and three (43%) displayed progressive disease (PD) [93]. In keeping with that, lack of response to osimertinib and immediate progression have been described in a patient with G719S/T790M co-mutations [92] and another one with co-existing G719S, S768I, and T790M mutations [94]. Collectively, these data seem to indicate that osimertinib, as opposed to afatinib, is less effective in patients with *EGFR* G719X and other uncommon mutations than in those with classic *EGFR*-mutants, both in the presence and absence of T790M co-mutation.

In our cohort, we identified a case that carried the G719C/S768I combination and somehow surprisingly showed OR to erlotinib, considering that it also harbored *MET*-amplification, MET-overexpression, and mutated *TP53*. Similarly, Hong et al. observed PR to erlotinib in patients, whose *EGFR*M+ NSCLC harbored G719X or L861Q together with co-mutations in other cancer-driver genes [52]. Moreover, Lund-Iversen et al. [79] reported one G719X/S768I co-mutated case showing PR to erlotinib for more than 14 months, while a long-lasting response to erlotinib with 9-year survival has recently been observed in a patient with NSCLC concomitantly harboring *EGFR* G719S and a *KRAS* G12C mutations [95]. Thus, given the apparently variable response of TKI-treated cases with uncommon mutants (alone or combined) the exact prognostic and predictive role of these mutations in NSCLC treated with different EGFR-TKIs remains to be further investigated.

A separate *EGFR*M+ NSCLC in our cohort exhibited the unusual combination of two rare exon 19 mutations, the microdeletion E746_R748del and the substitution A750P, together with the p.T1010I point-mutation in the *MET*-gene [51]. The response of these two exon 19-mutations to EGFR-TKIs is insufficiently determined, while the *MET*-substitution has been associated with decreased sensitivity to these drugs [96]. Nevertheless, our case did show PR to erlotinib with PFS longer than 17 months. Another case in our cohort displayed an insertion in *EGFR* exon 19 resulting in the 6-amino-acid duplication I744_K745insKIPVAI together with a missense *TP53*-mutation and increased *MET*-gene copy number associated with MET-overexpression. The sensitivity of *EGFR* exon 19-insertions (exon 19ins) to EGFR-TKIs is unclear, given that these mutations have been observed in only 0.26% and 0.11% of large Caucasian and Asian cohorts of *EGFR*M+ NSCLC patients, respectively [97,98]. This probably reflects not only a rare occurrence, but also the fact that probes for *EGFR* exon 19ins and the exon 20 insertion A763_Y764 insFQEA (see underneath on page 11 in this Section 2.1) are not always incorporated in the commercially available targeted mutation testing kits, thus higher frequency of these and other uncommon *EGFR*-mutations might be expected to be recorded with the increasing use of targeted NGS, whole-exome sequencing and whole-genome sequencing [98]. Recently, a meta-analysis of the few published cases with exon 19ins indicated that these mutations were associated with slightly lower RR than patients with common *EGFR*-mutations (56% vs. >65%) and a median time to progression of 10.4 months, but incomplete PFS/OS data in this small cohort hampered the comparison [98]. In this regard, our case with the exon 19 I744_K745insKIPVAI mutation showed no OR to erlotinib, however one cannot exclude that this was partly or completely due to the concurrent *TP53*-mutation and increased *MET-*gene copy number [51].

Additional uncommon somatic *EGFR*-mutations that have been detected in NSCLC patients displaying very rapid disease progression after initiation of first line TKI-treatment are the L747P substitution in exon 19 and short in-frame insertions/duplications in exon 20. The very rare L747P seems capable of conferring intrinsic resistance to EGFR-TKIs of all three generations [54,99,100,101], though the mechanism is still unclear. Another very uncommon mutation at the same position of EGFR, L747S, has sporadically been observed both as secondary TKI-resistant mutant in the setting of acquired TKI-resistance [57,58,102] and as de novo mutation in cases with a co-existing classic sensitizing *EGFR*-mutation, like L858R, not responding to 1G EGFR-TKIs [54,86].

In-frame exon 20ins represent 5–10% of all *EGFR*-mutations in NSCLC and occur more frequently between codon 767 and 775 encoding the C-helix of EGFR-TK domain (A767 to C775) that regulates the binding of both ATP and EGFR-TKIs. They are though, a heterogeneous group of mutations with >50 different insertion types reported and spanning a significantly wider stretch of exon 20 [103]. Patients with exon 20ins display primary resistance to EGFR-TKIs of 1G/2G with reported RR and median PFS of <10% and 1–3 months, respectively [1,79,83,86,87,103]. The crystal structure and cell-based mutation screening of exon 20ins suggest that these mutants have unchanged ATP-binding pocket but, unlike sensitizing mutations, they activate EGFR by changing the conformation and relieving key autoinhibitory interactions within the C-helix of the TK-domain, which, in turn, sterically diminish the access of the TKIs to EGFR drug-binding pocket [103,104]. The EGFR A763_Y764insFQEA in-frame insertion, which accounts for 8–11% of all exon 20ins mutants and structurally and enzymatically more closely resembles L858R than other exon 20ins, is an exception as both preclinical and clinical data indicate that it is sensitive to erlotinib, gefitinib and afatinib [98,103]. Accordingly, the analysis of patients harboring the A763_Y764insFQEA insertion displayed a RR to EGFR-TKIs of 73% [98].

The effect of osimertinib on EGFR exon 20ins appears controversial. NSCLC-derived cell lines and Ba/F3 cells that were transduced with clinically relevant exon 20 insertions know to be associated with resistance to 1G/2G EGFR-TKIs, such as Y764_V765insHH, A767_V769dupASV, and D770_N771insNPG, showed comparable sensitivity to afatinib and osimertinib. Both drugs were significantly more effective in inhibiting the growth of these cells than erlotinib, but osimertinib exhibited greater potency and mutation-specificity than afatinib [92]. On the other hand, another recent in vitro study has shown that EGFR-TKIs of all three generations were unable to hinder common EGFR exon 20ins mutants, when used in concentrations not affecting the wt EGFR [105]. Although, single clinical cases and structural studies suggest that some exon 20ins may indeed respond to osimertinib [104,106], the efficacy of this drug on these mutations at approved or higher dosage remains to be substantiated by additional dose-adjusted clinical studies and awaits the results of specific ongoing trials [107]. Recent preclinical data have shown that the combination of afatinib or osimertinib with the anti-EGFR monoclonal antibody cetuximab may inhibit the growth of NSCLC cells carrying certain types of exon 20ins in vitro or in a xenograft mouse model [108]. Although skin toxicity is a substantial limiting factor for the clinical application of this combined treatment, recently PR was reported with the usage of afatinib + cetuximab in three of four NSCLC patients with EGFR exon 20ins receiving this therapeutic combination [109,110]. Moreover, new selective TKIs targeting *EGFR* and *ERBB2* exon 20 insertions, such as poziotinib, TAS6417, and others have shown efficacy in preclinical models and promising preliminary results in early clinical trials. This mechanistically can be ascribed to their smaller size and higher level of halogenation and flexibility, as compared to the larger and more rigid TKIs of 1G/2G [111,112]. A potentially alternative therapeutic approach considers that EGFR exon 20ins mutants depend on the association with the heat shock protein 90 (Hsp90) chaperone system. Accordingly, the Hsp90-inhibitor luminespib has recently shown inhibitory activity against NSCLC cells with different *EGFR* exon 20ins and OR in a patient with LAC carrying an exon 20ins resistant to EGFR-TKI treatment [105].

In addition to somatic mutations, other reported EGFR-associated mechanisms for inherent resistance to EGFR-TKIs are the germline T790M polymorphism in exon 20 and the germline V843I mutation in exon 21 [113,114,115]. NSCLCs with germline T790M or V843I mutations are predominantly LACs harboring a secondary somatic classic *EGFR*-mutation and occur more frequently in females and non-smokers [115]. The families harboring the T790M or V843I mutations are predisposed to NSCLC development as these mutations contribute to tumorigenesis by promoting phosphorylation of EGFR and its downstream signaling proteins. Like T790M, the V843I mutation is associated with familial clustering of NSCLC and appears to provide resistance to EGFR-TKIs through structural modification of EGFR that sterically hinders TKI binding [113,114]. Thus, cases with germline T790M or V843I mutations could be categorized as a class of familial lung cancer syndrome with resistance to 1G/2G EGFR-TKIs but possibly sensitive to 3G TKIs [114,115].

Therapeutic strategies for uncommon *EGFR*-mutations are limited by the low incidence and heterogeneity of these alterations, which limit their inclusion in most clinical trials for EGFR-TKI-based treatment. Thus, the evidence regarding uncommon *EGFR*-mutations, until now, has relied on single case reports or small case series. Studies of a larger scale are warranted [81]. A summary of de novo *EGFR-*mutations and -co-mutations that have been associated with reduced response/intrinsic resistance to EGFR-TKIs is presented in Table 1.

### 2.2. Role of Co-Mutations in Alternative Cancer-Drivers

Several studies have addressed whether possible co-mutations in alternative cancer-drivers could represent mechanisms of inherent resistance to EGFR-TKIs. An exploratory investigation by targeted NGS of 197 consecutive NSCLCs with sensitizing *EGFR*-mutations displayed 11 cases intrinsically resistant to EGFR-TKIs, but the authors were able to detect concomitant driver mutations in only three of them (one case showed *EGFR* T790M mutation, one *MET*-amplification, and one *ALK*-fusion) [116]. In the eight cases without detectable driver co-mutations, primary resistance may have been caused by DNA-mutations or other events (RNA splicing variants, epigenetic mechanisms, protein modifications, pharmacokinetic factors) not assessable by the utilized NGS panel. In our cohort of erlotinib-treated NSCLCs, 71% of cases revealed concurrent mutations in alternative cancer-drivers prior to TKI-treatment [51]. In 67% of these cases, we identified *TP53*-mutations, while 60% of them carried co-mutations in either *MET*, *KRAS*, *NRAS*, *SMAD4*, *PIK3CA*, *CTNNB1, DDR2, ERBB4*, *FGFR1*, or *FGFR3*. Other analyses of gefitinib- and erlotinib-treated *EGFR*M+ NSCLC cohorts using the same targeted NGS platform as ours showed co-mutations in analogous genes and at very similar frequency [52,53,117,118]. Importantly, overall the TKI-receiving patients harboring co-mutations displayed a poorer OR than those without co-mutations [51,52,53,117,118]. Likewise, a large database-study assessing characteristics and outcomes of NSCLC patients carrying multiple molecular alterations showed that cases with *EGFR/KRAS* and *EGFR/PIK3CA* co-mutations were associated with shorter PFS during TKI-treatment than patients with only *EGFR*-mutations [44]. Finally, a recent investigation of 374 consecutive untreated metastatic *EGFR*M+ NSCLCs undertaken by the wide-targeted NGS platform used at the Memorial Sloan Kettering Cancer Center (MSKCC) in New York found 200 cases with coexisting alterations, the most frequent of which were mutations in *TP53, PIK3CA, CTNNB1*, and *RB1* and focal amplifications in *EGFR, TTF1, MDM2, CDK4*, and *FOXA1* [38]. Importantly, amplification of *ERBB2* or *MET* or mutation in *TP53* were significantly associated with a shorter time to progression [38]. Together, these studies suggest that in untreated advanced *EGFR*M+ NSCLC co-mutations in other cancer-drivers are much more frequent than previously anticipated and may act as mechanisms of inherent resistance to gefitinib and erlotinib. Yet, when analyzed more in detail, the contribution of each of the mutations that have been implicated in primary TKI-resistance is not always clear-cut.

#### 2.2.1. Alterations in the TP53 and RB1 Tumor-Suppressor Genes

The co-mutations most frequently detected by widely applied targeted NGS-assays in this setting are those in the tumor suppressor gene *TP53.* These mutations are known to occur in over 50% of LACs in Caucasians and with lower frequency in East Asians [3,6,7,40]. Mutations in *EGFR* and in *KRAS* usually occur in the founder clones of LAC (most frequently in non-smokers and smokers, respectively), whereas *TP53*-mutations frequently appear during advanced stages of tumor development, indicating that they play a role during tumor progression rather than initiation [7,11,12,119]. Several *TP53* mutants have been reported to contribute to acquired TKI-resistance by interfering with the TKI-mediated cell-cycle arrest and apoptosis [120,121,122,123]. Yet, with respect to intrinsic TKI-resistance, several reports have shown only a marginal, not always significant, negative effect of *TP53* co-mutations on the OR of gefitinib- or erlotinib-treated *EGFR*M+ NSCLC-patients [51,52,53,117,118]. This lack of significant association between co-existing *TP53*-mutations and sensitivity to TKIs may be ascribed to stochastic variations related to relatively few observations and/or the type of *TP53*-mutations identified in these studies that may differently interfere with the effect of TKIs. Indeed, analyses of larger cohorts of pre-treatment *EGFR*M+ LAC samples not only confirm that *TP53* mutations are among the most frequent (>50%) concomitant alterations in this cancer type [12], but also show that they are associated with significantly faster tumor progression after treatment with EGFR-TKIs of all three generations [38,124]. Thus, co-mutations in *TP53* may represent a mechanism of intrinsic TKI-resistance, though the role of different types of *TP53*-mutations remains to be elucidated. Moreover, inactivation of *TP53* function in *EGFR*M+ NSCLC may also occur post-transcriptionally via another frequent primary co-alteration, i.e., de novo amplification of the *MDM2* oncogene, which results in inhibition of the p53 protein [38] and is associated with worse PFS during TKI-treatment with osimertinib [124].

Recurrent inactivation of retinoblastoma protein 1 (RB1), another major tumor suppressor and cell-cycle regulator downstream EGFR, has also been detected in LAC, either due to mutation of the *RB1* gene itself, or deletion/mutation/methylation of other cell cycle-related tumor suppressor genes, such as *CDKN2A,* or mutation/amplification of cell cycle-inducing proto-oncogenes, such *CCND1/2, CCNE1, CDK4/6* [3,10,12,40]. Therefore, lack of cell-cycle control can potentially represent a major hurdle to the therapeutic effect of EGFR-TKIs in NSCLC. In this regard, recent studies by Yu et al. and Kim et al. [38,124] identified *RB1*-mutations among the most common concurrent alterations in TKI-naïve *EGFR*M+ NSCLCs. Moreover, co-mutations in *RB1* were a predictor of much faster progression following therapy with EGFR-TKIs (median PFS, 1.9 vs. 11.7 months; *p* < 0.001; multivariate analysis showing HR = 5.6) [124]. Relatedly, Blakely et al. identified in cfDNA of patients with advanced *EGFR*M+ NSCLC co-alterations of cell cycle genes, such as *CCND1/2*, *CCNE1*, *CDK*4/6 that are all coding for functional inactivators of the Rb1-protein. The co-mutation or -amplification of these genes were significantly associated with poor response to EGFR-TKIs in these patients [12]. Investigations of additional large cohorts of *EGFR*M+ NSCLCs at baseline using comprehensive gene panels may allow to further define the role played in intrinsic TKI-resistance by co-mutated genes in the p53- and Rb-pathways. This is particularly important, since alterations of these two major tumor suppressor pathways are not only frequent in NSCLC, but also remain among the least therapeutically actionable events in this disease [3,7,10].

#### 2.2.2. ALK- and ROS1-Fusions

Among pre-treatment alterations in protooncogenes that could affect the initial response to EGFR-TKIs, those in *ALK, ROS1* and *MET* are of interest not only mechanistically, but also because of the availability of ALK-, ROS1- and MET-targeted drugs. We did not find any *ALK*-rearrangement or ALK-fusion protein expression by FISH and IHC in our cohort of *EGFR*M+ NSCLCs [51]. At a first glance, this is consistent with the fact that *EGFR*-mutations and *ALK*-fusions have been largely described as mutually exclusive in untreated NSCLC and as mutual causes of acquired resistance to ALK-TKIs and EGFR-TKIs, respectively [17,18,43]. However, co-existing *EGFR*-mutations and *ALK*-rearrangements have been reported in a small but relevant number of NSCLC patients (reportedly from 0.09% to 1.6% of all NSCLs) and a prevalence ranging from 0.5% to 4% of *EGFR*M+ NSCLCs and from 4.4% to 19% of *ALK*-rearranged NSCLCs (highest in East Asian patients), depending on the study and utilized detection methods [3,42,45,125,126,127,128]. These studies have also indicated that deep NGS sequencing analysis significantly augments the detection rate of the co-alteration in TKI-naïve NSCLC as compared to less sensitive methods such as PCR, Sanger sequencing and FISH.

Intra-tumoral clonal heterogeneity, co-existence of the two alterations in the same tumor cells, very rapid acquisition of the co-alteration right after initiating TKI-treatment, or a combination of these circumstances have been envisioned as possible causes of *EGFR/ALK* co-alteration in NSCLC [42,91,128]. Also compatible with all these possibilities is the reported detection of cases with concurrent *EGFR/KRAS* co-mutations and *ALK*-rearrangement [44,45,129]. A literature review of 100 NSCLC cases with concomitant *EML4-ALK*-rearrangement and *EGFR*-mutation has recently been published [91]. Yet, the effect of co-existing *ALK*-fusions on the response to first-line EGFR-TKIs has not been fully clarified. Single case reports have shown conflicting results, as reviewed by Yang et al. [128] and Lo Russo et al. [91]. In a large Chinese cohort of 977 screened NSCLC patients, four out of 13 of the cases identified with *EGFR/ALK* co-alterations responded only to either an EGFR-TKI or an ALK-TKI at different time points, suggesting that one of these oncogenes might have had a dominant impact in these four cases [128]. Moreover, no significant differences in median OR to first-line EGFR-TKIs between *EGFR/ALK* co-altered cases and *EGFR*-mutant alone was reported (RR of 80% (8/10 pts.) vs. 66% (55/84 pts.), median PFS of 11.2 vs. 13.2 months, median OS of 18.5 months vs. 21.3 months, respectively), suggesting that the benefit of TKIs was comparable in the two groups [125,128]. Similarly, Ulivi et al. [126] observed clinical benefit of first-line EGFR-TKIs in 67% (4/6) of patients with double *EGFR*/*EML4-ALK* mutations vs. 81.8% of patients with only *EGFR*-mutations at baseline. In contrast, Won et al. treated three patients with concomitant *EGFR*-mutation and *EML4-ALK* fusion with gefitinib and observed PD in two and SD with PFS of 6 months [127]. This was opposed to the good response in the eight patients they treated with ALK-TKIs that exhibited RR of 88% (7/8 with PR) and prolonged PFS [127]. The intratumoral heterogeneity of *EGFR*-mutations and *ALK*-fusions might be a possible explanation for the variable efficacy of EGFR-TKIs in *EGFR/ALK* co-altered patients [91,130]. In addition to the relative abundance of *EGFR*-mutations and *ALK*-rearrangements, the levels of phosphorylation of EGFR, ALK, or downstream proteins detectable in tumor samples by IHC have been proposed for predicting the efficacy of TKIs in NSCLC with *EGFR/ALK* co-alterations [125,128]. However, this needs to be further validated in additional cases. In their review of 100 published cases with *EGFR/ALK* co-alteration, Lo Russo et al. [91] described that 43.4% of those treated with EGFR-TKIs showed an OR vs. 51.3% of those treated with ALK-TKIs, while of those sequentially treated with EGFR- and ALK-TKIs, 23.1% responded to EGFR-TKIs and 42.3% subsequently responded to ALK-TKIs. Thus, ALK-TKIs seem to be slightly more effective than EGFR-TKIs in patients with concomitant *EGFR*- and *ALK*-alterations, but the reasons for the variable response to EGFR- and ALK-TKIs in these patients remain to be defined [91]. Larger multicenter-studies would be necessary to better understand the responsiveness to TKIs of NSCLC with *EGFR/ALK* co-alterations, as the available data, despite constantly growing, are based on few and inconsistent case reports that do not allow to draw definitive conclusions.

As for *ROS1*, comprehensive studies of metastatic NSCLC including cases with *ROS1*-fusions have shown conflicting results in terms of concomitant oncogenic driver mutations. Wiesweg and coworkers detected *ROS1*-fusions in almost 5% of cases in a large cohort of 805 patients with metastatic LAC and 36% of these *ROS1*-positive cases presented with concomitant oncogenic driver mutations [131]. These included co-mutations in *EGFR*, *KRAS*, *BRAF*, or *PIK3CA*, with the most frequent ones being those in *EGFR*, identified in six patients and showing variable response to EGFR-TKIs in the five patients treated with these drugs. In contrast, Lin et al. detected very few concurrent alterations in other oncogenic drivers, especially no *EGFR* co-mutations, in a cohort of 62 patients with *ROS1*-positive NSCLC [132]. Moreover, by assessing an independent data set of 166 *ROS1*-rearranged NSCLCs detected by FoundationOne CDx test (Foundation Medicine), these authors only identified one case with concomitant driver mutation in *EGFR*. Thus, further studies are necessary to evaluate the possible impact of *ROS1* co-alterations on the response to TKIs in *EGFR*M+ NSCLC. Given the quite rare occurrence of *ROS1*-fusions in NSCLC, it is predictable that most data on this issue will be provided by case reports.

#### 2.2.3. MET-Alterations

In NSCLC cells uncontrolled activation of the signaling induced by the hepatocyte growth factor (HGF) and its receptor, the Mesenchymal-Epithelial Transition factor (MET), can be triggered by increased HGF levels, receptor overexpression due to *MET*-amplification or post-transcriptional modifications, point-mutations of *MET* TK-domain and other functional domains, or reduced MET-degradation due to *MET* exon 14 splicing-site mutants resulting in exon 14 skipping/deletion. The consequent abnormal MET-signaling can promote proliferation, survival, migration, invasiveness, and EMT of NSCLC cells [133]. *MET-*alterations (especially amplification) have been reported in 5–20% of NSCLCs with acquired resistance to EGFR-TKIs, representing approximately 5% of the cases treated with 1G/2G EGFR-TKIs and 20% of those receiving osimertinib [17,18,25,29,36]. Given that these tumors often remain dependent on EGFR-signaling, combining MET-inhibitors with continued EGFR-TKI treatment is considered a more effective strategy against them than switching from EGFR- to MET-inhibition alone [133,134].

MET receptor overexpression alone can induce malignant cellular transformation in vitro and in vivo, is detectable in approximately 50% of all patients with NSCLC and is a negative prognostic factor in NSCLC. However, MET overexpression in *EGFR*M+ NSCLC is not automatically associated with poor response to EGFR-TKIs, nor is an optimal predictor of response to MET-TKIs, as clinical responses to these drugs in NSCLC patients have been unsatisfactory in the absence of *MET-*mutation or -amplification [133,134]. Overall, the published data on MET expression in NSCLC suggest that this parameter, as assessed by IHC, does not necessarily reflect activation of MET-signaling and tumor MET-dependence [134]. Hence, evaluation of MET status by IHC remains a heterogeneous, suboptimal, and controversial predictor of response to TKIs, especially those against MET itself. This is also due to the lack of standardized methods for performing MET IHC (different sensitivity/specificity of the various commercial antibodies against different epitopes of MET) and for scoring MET expression levels [134]. These issues were illustrated also by a recent phase Ib/II study combining the selective MET-TKI capmatinib with gefitinib in the treatment of *EGFR*M+ NSCLC patients that had acquired resistance to EGFR-TKIs associated with MET-dysregulation [135]. Only the highest MET expression by IHC (i.e., 3+) was predictive of response in this study and the ORR for the MET-overexpressing 3+ cases was 32%, thus noticeably lower than the ORR of >50% observed when targeting selected patient subpopulations harboring other NSCLC-drivers such as *EGFR-, ALK-, ROS1*- or *BRAF*-mutants [135]. Although MET IHC data are generally related to *MET*-amplification, biomarker data from clinical studies have yet to elucidate the connections of MET-overexpression with *MET*-mutation or -amplification as predictive biomarkers and indicators of NSCLC dependence on MET-signaling [134]. For these reasons, direct evaluation of increased *MET*-gene copy number amplification is currently preferred for assessing MET-addiction of tumors and predicting responses to TKIs [133,134].

Earlier studies identified de novo *MET*-amplification in approximately 3% of patients with *EGFR*M+ NSCLC as possible mechanism of intrinsic resistance to erlotinib and gefitinib [136]. In agreement with more recent findings in the general NSCLC population and in the subset of *EGFR*M+ LACs [12,46], our *EGFR*M+ NSCLC cohort displayed an overall frequency of *MET* copy number gain of 22% and high concordance between *MET*-amplification and MET-overexpression, though we also observed a few cases with MET-overexpression not associated with gene amplification [51], which is a relatively frequent event in NSCLC [46,133]. In addition, 60% of our patients with *MET*-amplification and/or MET-overexpression also carried a *TP53*-mutation, indicating a potential growth advantage for NSCLCs with co-existing disruption of EGFR-, MET- and p53-dependent signaling pathways. Preclinical models have demonstrated that *MET*-amplification promotes proliferation and survival of *EGFR*-mutant, TKI-treated NSCLC cells by activating both the MAPK and PI3K/AKT signaling as well as inhibiting the proapoptotic proteins BIM and APAF-1 [137,138,139]. In the clinical setting, a significant fraction of cases with acquired resistance to EGFR-TKIs are associated with *MET*-amplification (around 3% of those receiving 1G/2G TKIs and up to 20% of osimertinib-treated ones), which is likely due to clonal selection of preexisting *MET*-amplified cells during TKI-treatment [17,18,25,29,36,133,139]. Supporting this notion, *MET*-amplified cell subpopulations have been identified at low frequencies (reportedly representing <1% of tumor cells) in pre-treatment specimens from cases that subsequently exhibited *MET*-amplification as main mechanism of resistance at disease progression, thus indicating that dominant clones had emerged from the preexisting cells under TKI-induced selective pressure [55,139].

Although the involvement of *MET* in the acquired TKI-resistance is well recognized, the potential role played by this gene in the primary TKI-resistance appears less clear. In addition to our series of NSCLCs with *MET* co-alterations, single cases of *EGFR*M+ NSCLC with concurrent de novo *MET*-amplification, inherent resistance to EGFR-TKIs, and response to the subsequent dual EGFR/MET blockade by the combination erlotinib/crizotinib have been described [140,141]. Similarly, a Japanese group retrospectively detected *MET* copy number gain at baseline in 11 out of 35 gefitinib-treated *EGFR*M+ LACs and showed that this event was associated with a high risk of progression and death (HR of 3.83 and 2.25, respectively) [142]. In keeping with that, the recent broad analysis of untreated *EGFR*M+ NSCLCs performed at the MSKCC showed that concomitant *MET*-amplification correlated with shorter time to progression on first-line EGFR-TKI with a HR of 3.7 [38]. Supporting the importance of MET signaling in primary resistance to TKIs, another Japanese study detected high-level expression of the MET-ligand HGF in 29% of NSCLC patients inherently not responding to EGFR-TKIs [143]. Interestingly, in this study high-level HGF expression turned out to be more frequently associated with intrinsic and acquired EGFR-TKI resistance than *EGFR* T790M mutation or *MET*-amplification [143]. Collectively, the data indicate that concurrent activation of MET-driven bypass signaling at baseline in *EGFR*M+ NSCLC is an event capable of immediately interfering with the efficacy of EGFR-TKIs but can also represent a potential therapeutic co-target for combinatorial first-line strategies aimed at overcoming EGFR-TKI resistance. The above-mentioned phase Ib/II trial combining gefitinib with the selective MET-inhibitor capmatinib has shown OR in a substantial fraction of *EGFR*M+ NSCLCs acquiring resistance to the EGFR-TKI through increased *MET*-gene copy number (ORR of 47% in cases with six or more mean *MET* copies/cell as determined by FISH), thus confirming the clinical feasibility and usefulness of concomitant blockage of EGFR- and MET-signaling in tumors with *EGFR*/*MET* co-alterations, at least in the progression setting [135]. Other new selective MET-inhibitors, such as volitinib, savolitinib, and tepotinib, are currently being tested together with EGFR-TKIs in phase I/II trials for patients with advanced NSCLC [133,134].

However, in our cohort the presence of altered *MET*-status at baseline did not inevitably result in a lack of OR to erlotinib-treatment [51]. The above-mentioned case with co-existing *EGFR* exon 19-duplication (I744_ K745insKIPVAI), *TP53*- mutation, and increased *MET* copy number associated with MET-overexpression, did not respond to erlotinib [51], conceivably reflecting a so-called polyclonal TKI-resistance [57]. In contrast, other cases with *MET*-mutation or copy number gain and/or MET-overexpression, did show a PR to erlotinib, regardless of the co-presence of a *TP53*-mutation. Similarly, others recently reported in a cohort of 133 *EGFR*M+ NSCLCs, four cases having co-mutation of *MET* and yet displaying OR to 1G EGFR-TKIs [53]. Thus, despite *MET*-amplified tumor cells potentially resistant to EGFR-TKIs may already exist at baseline, the clinical significance of these cells in intrinsic resistance requires further confirmation in large cohorts. Ideally, these future studies should also establish the most efficient MET-biomarkers (IHC, FISH, and DNA/RNA sequencing), since part of the above-mentioned discrepancies regarding OR to TKIs in *MET* co-amplified cases could be due to the lack of standardized methods for determining *MET*-amplification. In particular, the *MET*-gene copy number gain required to induce clinically significant MET-overexpression and ligand-independent activation remains poorly defined [49,133,134]. This reflects the fact that traditionally *MET*-amplification has been identified in routine clinical practice by FISH and categorized in low- and high-level amplification, with some reports additionally including also intermediate-level amplification, based on different *MET*-to-chromosome seven centromere (*MET*:*CEN7*) ratios and/or *MET* copy number per cell that slightly vary from study to study [46,49,51,133,134,144,145]. In this respect, the *MET:CEN7* ratio is considered by many as parameter reflecting true gene-amplification, whereas the *MET* copy number per cell is affected by amplification of the gene or of a chromosomal region, or by polysomy. Co-alterations in other oncogenic drivers such as *EGFR, ALK, ROS1, KRAS, BRAF, ERBB2*, and *RET* have been reported to occur much more frequently in NSCLCs with low-/intermediate-level *MET*-amplification than in cases with high-level amplification, suggesting that *MET* is the main driver in the latter tumors [31,144,145]. However, *EGFR*M+ NSCLCs with co-existing high-level *MET*-amplification at baseline do exist [38,46,51,140,141,142,144], suggesting the possibility that in these cases heterogenous clones with either mutated *EGFR* or amplified *MET* might be present. In this respect, in a recent cohort of 200 consecutive patients with treatment-naïve metastatic *EGFR*M+ assessed by FISH, 52 (26%) patients displayed concomitant *MET*-high (defined as copy number gain of 5 or greater) at diagnosis. In 46 cases (23%) this was due to polysomy, while in the other six (3%), true amplification (defined by *MET:CEN7* > 2) was detected [145]. Notably, assessing the copy number gain did not correlate with the following response to 1G/2G EGFR-TKIs, as no significant differences in median time-to-treatment failure (TTF; 12.2 months vs. 13.1 months) and RR was found between *MET*-high and -low groups. In contrast, five out of the six patients with co-existing *MET*-amplification at baseline displayed substantially poorer response to EGFR-TKIs (TTF less than 6.5 months), with the two cases with the highest *MET:CEN* ratio rapidly progressing within the first month of treatment [145]. These data support the notion that *EGFR*M+ NSCLCs with assessed true *MET*-amplification at baseline respond poorly and progress very rapidly, thereby fulfilling the temporal criteria for primary resistance [15,145]. In contrast, cases with increased *MET*-gene copy number assessed by arbitrary thresholds, may not necessarily lack response to EGFR-TKIs. Whether they may or may not have an impact on PFS after EGFR-TKIs requires comparison with *EGFR*M+ cases without concomitant *MET*-alterations.

NSCLCs with high-level *MET*-amplification have shown significantly better response to MET-signaling inhibition than cases with lower levels of *MET*-amplification/copy number gain, both when increased *MET* copy number was the only reported oncogenic driver and in *EGFR*M+ NSCLCs with *MET*-dependent acquired resistance to EGFR-TKIs [49,134,135]. Moreover, *EGFR*M+ NSCLCs with concomitant high-level *MET*-amplification may inherently show poor response to EGFR-TKIs [38,142], but associating a blocker of MET-signaling to the treatment appears a promising approach for tackling the primary resistance to EGFR-TKIs in these cases [140,141]. Thus, standardized methods for identifying and classifying co-amplification of *MET* in *EGFR*M+ NSCLCs should be implemented for planning combinatorial therapies aimed at improving the outcome of cases with these co-alterations. Given that IHC-assessed MET-protein expression does not seem to accurately predict MET-induced resistance to EGFR-TKIs or sensitivity to MET-inhibitors in *EGFR*M+ NSCLC, and since it is still debated whether *MET:CEN* ratio is the best predictor for these drugs [133,134,135], alternative indicators of downstream MET-activation by increased *MET*-gene expression might be necessary. In this regard, MET-phosphorylation or MET protein overexpression together with increased *MET* copy number or the implementation of a MET-activation-dependent MET:GRB2 proximity ligation assay have been proposed [133].

*MET* exon 14 mutations (*METex14*) were detected in almost 3% of lung carcinomas of different histotypes, prevalently in elderly smokers, with highest frequency in adenosquamous carcinomas, sarcomatoid carcinomas with an adenocarcinoma-component, and LACs [146]. However, the incidence of *METex14* in LAC of East Asian patients without alterations in other driver-genes such as *EGFR, ALK, ROS1, KRAS* or *RET* appears significantly higher [147]. Until now, *MET*ex14 alterations have not been reported in association with acquired resistance to EGFR-TKIs in *EGFR*M+ NSCLC [134]. This may reflect the initial notion of *METex14* as mutually exclusive with other oncogenic driver-mutations prevalently occurring in non-smokers, such as those in *EGFR* or *ALK.* Nonetheless, concomitant amplification of *MDM2*, *CDK4*, *ERBB2*, or *EGFR*, or *KRAS*-mutations were observed in subsets of NSCLCs with *METex14* [145,148], which possibly signifies the co-existence of clones with different drivers. Thus, the role, if any, of *METex14* in primary resistance to EGFR-TKIs warrants future investigation.

#### 2.2.4. RAS-, BRAF, ERBB-, DDR2-Mutations

*KRAS*-mutations are one of the most common genetic events involved in the pathogenesis of LAC in which they are identifiable at a frequency of 20–30% of Caucasian patients and 2–10% of East Asian patients, particularly in smokers [3,6,7]. Most *KRAS-*mutations in NSCLC are seen in codon 12 and 13, but rarer mutations occur also in codon 61 and 146. These mutations can also emerge during treatment of *EGFR*M+ NSCLC with EGFR-TKIs and can cause secondary TKI-resistance to these drugs, given their capability of constitutively activating effectors downstream of EGFR [49,149]. We and others reported the existence of rare cases with co-mutation of *EGFR*- and *KRAS*-mutations in LACs prior to TKI treatment [3,43,45,47,51,52,126]. When treated with EGFR-TKIs, some of these *EGFR/KRAS* co-mutated cases showed PD or SD as best response, while others unexpectedly displayed a PR, even when they harbored additional driver-mutations such as *TP53*-mutations [45,51]. On the other hand, Oxnard et al. studying acquired resistance in osimertinib-treated NSCLCs with secondary T790M mutation, observed that in contrast to the patients maintaining T790M at the time of resistance (32%) and progressing after approximately 15 months of treatment mainly by acquisition of tertiary C797S mutation, the patients who had lost T790M (68%) progressed within 6 months through a range of competing resistance mechanisms, including *KRAS*-mutations and targetable gene fusions [32]. Together, these data suggest that pre-existing resistant clones with these alterations are selected and expanded by TKI-treatment, ultimately leading to resistance acquisition over relatively short time, but they may not necessarily cause immediate inherent resistance [32,45,51]. Others recently noticed poorer OR and shorter PFS (<3 months) in patients with high relative AF of *KRAS*-mutants vs. sensitizing *EGFR*-mutations as compared to those with low AF [53]. Thus, to explain the variable responses of *EGFR/KRAS* co-mutated patients, the authors inferred that KRAS-induced intrinsic resistance may ensue only when the relative AF of *KRAS*-mutants is sufficiently high to counteract the effect of EGFR-TKIs on cancer cells [53], a concept that in principle may be applied to other resistance-associated co-mutations. Another potential explanation may come from the recent study by Moll et al. [150] suggesting that, in contrast to common opinion, resistance to 1G TKIs in *KRAS*–mutated NSCLC may not be entirely caused by constitutive activation of KRAS but also by transcriptional upregulation of all the ERBB-family members. The authors also showed in cell lines and a mouse model that growth of *KRAS*–mutated NSCLC depends on upstream activation of EGFR. Consequently, genetical or pharmacological suppression of EGFR signaling by 1G EGFR-TKIs transiently down-regulates also the activity of mutant KRAS and related downstream signaling pathways. However, the gradual activation of the other ERBB-family members functions as a compensatory mechanism that can reestablish KRAS signaling over time and make cancer cells TKI-resistant [150]. In contrast, the pan-ERBB inhibitor, afatinib, can block this compensatory mechanism and stably inhibit KRAS activity, thereby reducing the growth of *KRAS*-mutated NSCLC cells in preclinical models [150]. Therefore, given the lack of effective therapeutic strategies against *KRAS*-mutated cancers, it might be of interest to test the capacity of afatinib alone or combined with other drugs to inhibit the growth of *EGFR/KRAS* co-mutated NSCLC in human patients.

Further illustrating the incompletely defined role of *RAS* genes in the complexity of inherent TKI-resistance, we observed also an *EGFR*M+ case that concomitantly carried mutations in *NRAS*, *TP53, ERBB4* and *DDR2* [51]. Although multiple, per se oncogenic mutations may imply polyclonal resistance, this case somehow surprisingly showed PR to erlotinib. Likewise, two cases with co-mutated *NRAS* at baseline reported by Rachiglio et al. showed OR to 1G EGFR-TKIs [53]. *NRAS*-mutations have been reported with a frequency of <1% in NSCLC, most commonly in association with adenocarcinoma histology and tobacco exposure, in analogy with *KRAS*-mutations [151]. However, in NSCLC *NRAS*- and *KRAS*-mutations not only display a distinct nucleotide transversion profile, but also a different position, in that 80% of *NRAS*-mutations affect codon Q61 and 20% codon G12, while >90% of *KRAS*-mutations occur in codon G12, 6% in codon G13, and only 2% in codon Q61 [151]. While *NRAS* and *KRAS* genes share conserved sequences, their protein products appear to regulate distinct oncogenic signaling events and to differently depend upon the downstream MEK pathway in NSCLC cells [151,152]. In this regard, the involvement of *NRAS*-mutations in TKI-resistance, despite being in principle comparable to that of *KRAS*-mutations, remains poorly explored. Interestingly, using TKI-resistant NSCLC cell lines, Eberlein et al. discovered that certain *NRAS* mutations and *NRAS* copy number gain are a frequent mechanism of resistance to erlotinib and osimertinib. Additionally, they showed in mouse models that combining osimertinib with the MEK-inhibitor selumetinib re-sensitized osimertinib-resistant *EGFR/NRAS* co-mutated lung tumors to this EGFR-TKI [153].

Sporadic cases of *EGFR*M+ LACS with co-mutations in *BRAF* and showing PD upon TKI-treatment have been reported by Hong et al. (1/58) and Rachiglio et al. (3/133) [52,53], suggesting that also *BRAF*-mutations may immediately interfere with the effect of EGFR-TKIs. However, the small number of analyzed patients precludes firm conclusions on the role of *BRAF*-mutations in intrinsic resistance to EGFR-TKIs.

In addition to the above-mentioned compensatory up-regulation [150], activation of parallel by-pass non-EGFR ERBB-signaling may also occur in TKI-treated NSCLC cells by alterations of *ERBB2/3/4* genes. *ERBB2*-amplification occurs in 1% to 10% of LACs, depending on stage, patients’ ethnicity and other mutations [3,12], and may represent an alternative mechanism of resistance to 1G EGFR-TKIs in T790M-negative patients [154]. Recently, *ERBB2*-amplification was reported to correlate with shorter time to progression on erlotinib (HR of 2.4) in a large cohort of *EGFR*-mutant NSCLCs [38]. It is also one of the EGFR-independent mechanisms of acquired drug-resistance observed in patients treated with osimertinib [25,29,30]. Mutations in *ERBB2,* similarly to those in *EGFR,* are more frequent in LACs of younger females and non-smokers. In Caucasians, up to 2% of LACs harbor *ERBB2*-mutants, whereas the incidence increases to over 8% in LACs of East-Asians [3,12,155,156]. *ERBB2*-mutations can affect the extracellular (exon 5-8) and the transmembrane (exon 17) domains but are much more frequent in the TK domain (exon 18-24), where, in analogy with *EGFR*-mutants, they can result in substitutions, exon 19 microdeletions, and in-frame exon 20 insertions/duplications [3,12,156,157]. The latter are the predominant *ERBB2*-mutation type in LACs and most typically are in-frame insertions of 3–12 bp between codons 775–881. The concurrent amplification of the mutated *ERBB2*-gene or the occurrence of *ERBB2*-mutations with other oncogenic drivers such as *EGFR*-mutations or *ALK*-fusions have rarely been observed in NSCLC prior to TKI-therapy and have been related to lack of sensitivity to the following treatment with 1G TKIs [3,155,156]. Although some clinical studies have indicated that *ERBB2*-insertions are intrinsically resistant to the pan-ERBB TKIs afatinib, dacomitinib and neratinib [157,158], a subset of *ERBB2*-substitutions and exon 20 insertions as well as *ERBB2*-amplification have displayed preserved sensitivity to these drugs [155,157,159,160,161,162]. This can be followed by acquired resistance through different mechanisms (*MET*-amplification, loss of *ERBB2*-amplification, EMT) [160]. Conversely, other preclinical studies and preliminary clinical results have shown that *ERBB2* exon 20 insertions/duplications may be sensitive to the selective EGFR/ERBB2 exon 20 inhibitor poziotinib, while they can cause resistance to EGFR-TKIs of all three generations. These studies also confirmed the heterogeneous inhibitory activity of neratinib on some of the insertions [111,163,164]. The recent “basket” trial SUMMIT for patients with advanced solid tumors harboring *ERBB2*- or *ERBB3*-mutations exhibited a very low RR to neratinib in the included NSCLC cases (*n* = 26, all with *ERBB2*-mutations), with PR confined to one NSCLC with a missense mutation in *ERBB2* TK domain, whereas no OR was seen in NSCLCs with *ERBB2* exon 20 insertions [157]. A clear tendency towards worse outcome was seen in the enrolled patients, whose tumors contained *ERBB2*-mutations co-existing with mutations in alternative RTKs (such as EGFR or ERBB3), members of the RAS/RAF/MAPK pathway or in *TP53* [157]. Cumulatively, these data suggest that *EGFR*M+ NSCLCs with concomitant de novo *ERBB2*-amplification or -mutations are very rare, but in case of occurrence, they may result in inherently poor response to EGFR-TKIs of all three generations.

Somatic *ERBB3*-mutations have low incidence (typically <1%) across solid cancer types such as NSCLC and the oncogenic effect of ERBB3 depends on dimerization with other ERBB-family members because of its very weak intrinsic TK activity. Thus, the role of *ERBB3*-mutations, if any, in primary response to EGFR-TKIs remains elusive. For instance, a case of advanced chemotherapy-resistant NSCLC, carrying the somatic V855A *ERBB3*-mutation homologous to L858R EGFR-activating mutation was reported, but its oncogenic effect in human and murine cell lines required concomitant overexpression of wt *ERBB2* [165], which per se can be oncogenic and thereby confounds these results. Even though preclinical studies like this and others have suggested that *ERBB3*-mutants may be oncogenic, no responses to neratinib have been observed in patients with *ERBB3*-mutated tumors (none were NSCLC) included in the SUMMIT trial [157]. Thus, the clinical impact of *ERBB3*-mutations as potential oncogenic driver and therapeutic target in NSCLC, is still unclear. Yet, overexpression of ERBB3 and activation of ERBB3 signaling has been observed in different types of human cancers, including NSCLC, in which these events have been related to drug resistance (including TKI-resistance), cancer progression and poor patient survival [166]. Earlier studies showed that *MET*-amplification, at least in part, causes resistance to 1G EGFR-TKIs in NSCLC by activating ERBB3 signaling, which could be mediated by a strong direct interaction of MET with ERBB3 [137,167]. Moreover, the ERBB3 ligand heregulin has been found overexpressed in a subset of NSCLCs, including also *EGFR*M+ cases refractory to 1G EGFR-TKIs [168,169]. Overexpression of heregulin makes *EGFR*M+ NSCLC cell lines resistant to erlotinib via sustained activation of the by-pass ERBB3-AKT signaling pathway and the growth of these cells can be inhibited by the pan-ERBB inhibitor afatinib or by combining erlotinib with the anti-ERBB3 monoclonal antibody patritumab [168,169]. Thus, the heregulin-ERBB3 axis is a potential alternative and pharmacologically revertible mechanism of intrinsic resistance to 1G EGFR-TKIs.

*ERBB4*-mutations reportedly occur in 1–8% of NSCLCs with higher frequency in patients of East-Asian ethnicity as for *EGFR*-mutations [3,6,7]. Some of the *ERBB4*-mutants identified in NSCLC are ERBB4-activating because crucially situated at the dimerization interfaces of the extracellular (Y285C and D595V) and TK (D931Y and K935I) domains and possess oncogenic properties [170]. The S239P *ERBB4*-mutation that we observed in our erlotinib-treated *EGFR/NRAS/TP53/ERBB4/DDR2* co-mutated case showing PR had not been previously reported in NSCLC [51]. It resides in the extracellular dimerizing domain of ERBB4 and has been described in esophageal cancer as an activating mutation [171]. Thus, it could potentially represent a bypass-mechanism linked to TKI-resistance, but the role of *ERBB4*-mutants in this process needs further investigation.

As for *DDR2,* this gene encodes the collagen discoidin domain receptor 2, a member of the discoidin subclass of the RTK protein family. Missense mutations of this gene are present in 4% of pulmonary SqCCs, in which they may represent a therapeutic molecular target [172]. *DDR2*-mutations are also occurring in approximately 1.5% of LACs (http://cancer.sanger.ac.uk/cosmic), though their frequency was reported increased to 16% in *EGFR*M+ NSCLC [118]. However, no clear oncogenic function or apparent impact on TKI-treatment of LAC has yet been identified [118,173]. Thus, the role, if any, of *DDR2*-mutations in TKI-resistance remains to be determined.

#### 2.2.5. PIK3CA- and PTEN-Mutations

Somatic mutations in the catalytic domain of *PIK3CA* are considered cancer-drivers and represent one of the mechanisms of acquired TKI-resistance, but they are also detectable in up to 3% of *EGFR*M+ LACs prior to TKI therapy [29,38,39,51,52,53]. Expression of *PIK3CA*-mutants in *EGFR*M+ NSCLC cell lines makes them resistant to EGFR-TKIs by activating AKT-signaling and inhibiting TKI-induced apoptosis [39]. The co-existence of *EGFR*- and *PIK3CA-*mutations has been associated with shorter median OS, suggesting synergistic activation of oncogenic pathways [29]. However, in retrospectively assessed cohorts of patients with advanced *EGFR*M+ NSCLC, the occurrence of *PIK3CA* co-mutations at baseline, despite being a negative prognostic factor, did not necessarily interfere with the effect of EGFR-TKI monotherapy in terms of RR, PFS, and duration of response [51,52,53,174]. Indeed, the reported *PIK3CA* co-mutated cases with allegedly acquired or intrinsic resistance to EGFR-TKIs often harbored mutations in other oncogenes or in tumor-suppressor genes that could be the actual cause of TKI-resistance [29,51,52,174]. Thus, the currently limited amount of data regarding *EGFR/PIK3CA* co-mutated NSCLCs does not allow to firmly conclude whether *PIK3CA*-mutations represent a mechanism of intrinsic resistance to EGFR-TKIs.

*PTEN-*deletions have been associated with acquired resistance to erlotinib and gefitinib [29]. A case with T790M mutation and a *PTEN-*deletion before osimertinib therapy, followed by lack of response and increase in the number of metastatic sites with *PTEN-*deletions during treatment was reported, suggesting possible multifocal *PTEN*-dependent intrinsic resistance to osimertinib [175]. However, only a limited number of genes was analyzed, therefore it cannot be excluded that baseline mechanisms other than *PTEN-*deletion could have contributed to this primary resistance [29]. More recently, co-mutations of *PTEN* have been associated with significantly shorter PFS in a Korean cohort of *EGFR*M+ NSCLC patients receiving osimertinib as second line following initial EGFR-TKI failure (2.6 vs. 10.3 months for cases without *PTEN* co-mutations; *p* = 0.001; HR = 5.8 in multivariate analysis) [124]. Thus, PTEN inactivation could represent a factor contributing to rapid progression on osimertinib.

#### 2.2.6. CTNNB1-Mutations

In our *EGFR*M+ NSCLC cohort we detected cases that prior to erlotinib treatment showed concomitant pathogenic mutations of the *CTNNB1* gene coding for β-catenin, the main effector in the Wnt/β-catenin signaling pathway that transactivates cell proliferation-related genes [51,176]. The recent wide studies of Blakely et al. [12] and Yu and coll [38] indeed confirmed that *CTNNB1-*mutations are common co-alterations in untreated advanced *EGFR*M+ NSCLCs, including cases with co-existing T790M, and that they are functionally active (able to activate cell signaling, proliferation, migration, and invasiveness). By longitudinal genomic analysis of liquid biopsies and tumor re-biopsies, Blakely et al. also identified *EGFR*M+ NSCLC patients with activating *CTNNB1* co-mutations already present in early tumor stages and subsequently persisting during progression to metastatic disease, which implied that these mutations were clonal and may play a co-pathogenetic role in *EGFR*M+ NSCLC [12]. Accordingly, preclinical data have indicated that *EGFR*-mutants can induce NSCLC development in part through upregulation and activation of β-catenin and that *CTNNB1*-mutations represent a potential downstream mechanism of acquired resistance to EGFR-TKIs [177,178,179]. Consequently, targeting the Wnt/β-catenin pathway might provide new opportunities for counteracting TKI-resistance [178,179]. However, these concepts and even more so whether co-mutated *CTNNB1* may play a role in primary TKI-resistance, await further clinical validation. In this respect, the NSCLC cases with concurrent *EGFR*- and *CTNNB1*-mutations that we identified partially responded to erlotinib-treatment [51].

#### 2.2.7. SMAD4-Mutations

Other concomitant mutations that we uncovered at baseline in our cohort of erlotinib-treated *EGFR*-mutant NSCLCs were in the *SMAD4*, *FGFR1*, and *FGFR3* genes [51]. The former encodes the SMAD4 transcriptional co-factor, which is a key player in TGF-β-mediated cell growth arrest, apoptosis, and antineoplastic function as well as EMT-induction [180,181]. Despite a study of the NSCLC genome showed a mutation rate of 4% among *SMAD*-genes [40], the incidence of inactivating *SMAD4*-mutations in *EGFR*M+ NSCLC has not been extensively studied and it remains poorly understood whether and how these mutations are implicated in intrinsic TKI-resistance. Co-presence of *SMAD4-*mutations has been observed in patients receiving gefitinib treatment, including cases that responded to this EGFR-TKI 117,118]. Blakely et al. detected by longitudinal genomic analysis of tumor-DNA and cfDNA from *EGFR*M+ patients *SMAD4* variants in both early resectable stage and metastatic stage, suggesting the clonal nature of these alterations. However, they detected the same frequency of *SMAD4*-mutations in a group of 20 osimertinib-responders and 21 osimertinib-non-responders [12], thereby casting doubts on the possible impact of these mutations on the response to EGFR-TKIs. Our patient with *SMAD4* co-mutation exhibited a mixed response to erlotinib [51]. Thus, further cases with *SMAD4* co-mutations need to be investigated to shed more light on their significance in TKI-resistance.

#### 2.2.8. FGFR-Alterations

Constitutive activation of the transmembrane protein FGFR1 by gene-amplification, -translocation or -mutation has been associated with various malignancies. *FGFR1*-amplification has been reported in up to 20% of pulmonary SqCCs and less frequently in LACs and SCLCs [182]. Single cases of *FGFR1*-fusions acquired during erlotinib- and osimertinib-treatment have also been observed [36,37]. Furthermore, preclinical and clinical investigations indicate that constitutively active FGFR1-signaling may represent a mechanism of acquired resistance to EGFR-TKIs [175,183,184]. Only few observations regarding co-mutations of *FGFR1* as a possible cause of primary TKI-resistance have been described. Lim et al. reported that two out of 20 *EGFR*-mutant NSCLC patients not responding to gefitinib harbored a concurrent *FGFR1*-mutation [118]. In contrast, we identified an advanced *EGFR*M+ case with co-mutations in the *FGFR1* and *TP53* genes, which nonetheless did show OR to erlotinib [51]. Thus, it is premature to conclude whether *FGFR1*-mutations may play a role in intrinsic TKI-resistance.

Activating *FGFR3*-mutations targetable by FGFR-TKIs have been initially described in subsets of urogenital cancers, but more recently oncogenic mutations affecting the extracellular and transmembrane domains of FGFR3 have also been identified in a minority of pulmonary SqCCs [185,186,187]. Moreover, a new study assessing by deep-sequencing and validating by mass spectrometry the spectrum of actionable alterations in LACs affecting patients of Indian origin has shown recurrent mutations of *FGFR3* TK-domain in 20/363 (5.5%) of cases [188]. These *FGFR3*-mutants were constitutively active and had oncogenic activity in vitro and in a xenograft mouse model, while both these effects were inhibited by FGFR-TKIs [188]. The *FGFR3*-mutated LACs occurred more frequently in younger patients and 25% of them concomitantly harbored *EGFR*-mutations [188]. In addition, oncogenic *FGFR3-TACC3* fusions have been detected in a small subset of advanced LACs, especially in cases with concomitant *EGFR*-mutations, in which the *FGFR3*-alterations appear to act as bypass-mechanism substituting for EGFR signaling and are associated with resistance to EGFR-TKIs of all three generations [36,37,189,190,191]. Most of these *FGFR3*-fusions emerged after treatment with different EGFR-TKIs, consistent with their involvement in acquired TKI-resistance, but given that often pre-treatment tissue was unavailable/insufficient for genetic testing in the investigations, one cannot exclude that FGFR3-signaling might also play a role in intrinsic resistance if it is already altered at baseline [36,37,190,191].

Indeed, we observed in our cohort of advanced *EGFR*M+ NSCLCs a case that prior to treatment concomitantly carried an activating *EGFR* exon 19del and a previously unreported 2 bp homozygous frame-shift microdeletion in *FGFR3* exon 17 resulting in elongated and structurally “deleterious”, highly pathogenic FGFR3 protein variant [51,68]. During first-line erlotinib-treatment this patient exhibited mixed response and serial tumor re-biopsies showed heterogeneous mechanisms of TKI-resistance occurring at different times and locations [68]. After only 7 weeks of therapy the patient developed metastatic pleural effusion, in which we detected transformation to SCLC that retained the *EGFR*- and *FGFR3*-mutations and partly responded to the following combination of carboplatin-etoposide and erlotinib-continuation. Instead, other pulmonary and hepatic metastatic sites still maintaining the *EGFR/FGFR3* co-mutations showed progression 6 months later associated with the appearance of the erlotinib-resistant T790M *EGFR*-mutation at very low AF. Intriguingly, the *FGFR3*-mutation persisted throughout tumor progression and at increasing frequency in the sequential biopsies taken at baseline, after the rapid pleural SCLC transformation, and when the new LAC-metastases appeared later during the treatment [68]. This case illustrated the complexity and heterogeneity of TKI-resistance mechanisms occurring in different progressive metastatic sites of *EGFR*M+ NSCLCs. Abnormal *FGFR3*-signaling might have contributed to the rapid progression in this patient despite erlotinib-treatment, with the phenotypic pleural SCLC transformation acting as an additional potent resistance mechanism that contributed to effectively by-passing the TKI-mediated EGFR-blockade. In this regard, *EGFR*M+ LACs transforming to SCLC with retained *EGFR*-mutation tend to downregulate the target EGFR protein, thereby becoming less sensitive to EGFR-TKIs and resembling SCLCs that typically express lower levels of EGFR than NSCLCs [68,192,193]. In contrast, in sites where SCLC transformation did not occur (possibly also prevented by the concomitant chemotherapy) the appearance of clones with T790M mutation could have ensured further progression together with the parallel constitutive FGFR3-signaling.

Collectively, our and others’ findings, support the notion that deregulated FGFR3-signaling represents an oncogenic driver in NSCLC and a potential mechanism of intrinsic and acquired resistance to EGFR-TKIs that may be reverted by FGFR-TKIs [37,51,68,188,189,190,191].

#### 2.2.9. Other Gene-Fusions

Actionable fusions affecting *RTK*-genes other than *ALK*- or *FGFR*-genes, such as *RET, NTRK,* and *EGFR* itself or involving *BRAF* have been identified as acquired resistance-drivers upon progression on EGFR-TKIs of all three generations, with higher frequency seen during treatment with osimertinib [32,35,36,37]. As speculated for the *FGFR3*-fusions, these gene-fusions apparently emerged after treatment with EGFR-TKIs, but because in several cases baseline samples were not available for genetic testing, the possibility that these alterations were pre-existing as intrinsically resistant clones cannot be completely excluded. In line with this notion, early resistance and rapid progression (within 6 months) on osimertinib in connection with the emergence of these fusions and loss of T790M was noticed in certain patients [32,36,37]. Importantly, the first clinical cases of *EGFR*M+ NSCLC with concurrent gene-fusions responding to the combination of EGFR-TKI and a specific inhibitor of the fused oncoprotein (e.g., ALK-, BRAF- or RET-inhibitor) are being reported, indicating the possibility of overcoming this mechanism of TKI-resistance by combinatorial therapy [35,36,37].

### 2.3. Phenotypic Changes

#### 2.3.1. Transformation to SCLC

Phenotypic transformation to SCLC or SqCC and EMT with change to sarcomatoid phenotype are mechanisms of acquired resistance to EGFR-TKIs of all three generations that can occur in up to 15% of *EGFR*M+ LACs during TKI-treatment and are associated with rapid clinical course [17,18,29,30,192,193]. The transformation to SCLC is the most common of these phenotypic changes and has been described in 3–10% of TKI-treated *EGFR*M+ LACs [17,29,192,193]. However, de novo *EGFR*M+ SCLC or mixed LAC-SCLC occasionally occurring in non-smokers independently of EGFR-TKI treatment and characterized by rapid progression have been reported too [192,193,194,195]. This raises the questions whether sometimes untreated disseminated *EGFR*M+ LACs may already contain a population of TKI-resistant SCLC cells as potential mechanism of inherent resistance and whether EGFR-TKI treatment can further select and expand this population giving rise to a genetically similar SCLC with acquired TKI-resistance [196]. Alternatively, LAC cells could just be forced to change their phenotype by TKI-treatment as adaptive change occurring immediately (intrinsic resistance) or gradually (acquired resistance) [196]. Although it can be difficult in routine clinical practice to establish whether the LAC-to-SCLC transformation is pre-existing or induced by the TKI- treatment [68], there is accumulating evidence for a dynamic molecular and cellular plasticity between LAC and SCLC, including the concept of a mutual origin from pluripotent alveolar cells [192,193]. In recent years, the advances concerning the biology behind the SCLC-transformation of *EGFR*M+ LAC have been substantial, whereas our understanding of the clinical course associated with this phenotypic change has been more limited, as clinical data have been obtained from case reports or small case series.

However, recent publications shed new light on these issues. One of these reports presented the hitherto largest retrospective multicenter study of *EGFR*M+ advanced lung cancers (*n* = 67) that either had phenotypically undergone the LAC-to-SCLC transformation upon TKI-treatment (*n* = 58) or were initially diagnosed as SCLC/mixed NSCLC-SCLC (*n* = 9) and considered as bona fide transformed LACs within a common biologic continuum [195]. Despite being of retrospective character, lacking standardized treatment and response evaluation, as well as uniform pathological analysis and genotyping of the historical samples (the patients had been treated between 2006–2018), this North American cohort, given its size, led to valuable conclusions on certain biological aspects, appropriate treatments, and prognostic implications for *EGFR*M+ LACs transforming to SCLC [195,197]. It also illustrated how clinically and genetically these transformed tumors represent a mixture of the features associated with *EGFR*M+ LAC and conventional smoking-related SCLC. The baseline demographics of the SCLC-transformed cohort [195] resembled those of the general population of patients with *EGFR*M+ LAC in terms of younger age, prevalent female gender, high representation of East-Asian ethnicity, and infrequent smoking habit of patients [1,2,90], though the percent of women and of never-smokers were slightly lower (57% and 73%, respectively). Also, the baseline distribution of founder *EGFR*-mutations was similar to that in *EGFR*M+ LAC in general [1], with strong prevalence of exon 19dels and L858R, which were detected in 69% and 25% of all patients, respectively while the remaining 6% harbored less common founder mutations, such as S768I, G719X or L861Q and two patients had an additional de novo T790M mutation. Importantly, all the SCLC-transformed cases did continue to harbor their original *EGFR*-mutation [195], as in previous reports of SCLC-transformation in TKI-treated LACs [68,192,193,198,199]. Some of these reports also indicated that following SCLC-transformation cancer cells became insensitive to EGFR-TKIs partly by downregulating the expression of EGFR protein and not by acquiring a secondary *EGFR*-mutation such as T790M [68,192,193]. In keeping with that, Marcoux et al. found that 15 of their 19 cases with previously detected T790M (two de novo and 17 acquired during TKI-treatment) had lost T790M after transformation to SCLC [195]. Collectively, these data are consistent with a separation of a T790M clone and a SCLC clone from a common founder LAC clone during the branching clonal evolution of *EGFR*M+ LAC described by Lee et al. [200]. They also suggest that the T790M clone may become dispensable for TKI-resistance after the phenotypic transition to SCLC [195,197,200], possibly because the EGFR protein downregulation represents a sort of “loss of TKI-target”.

Genotyping of Marcoux et al.’s cohort showed also significant incidence of *TP53-* and *RB1-*mutations in the LACs before undergoing SCLC-transformation and after having transformed as well as in the de novo *EGFR*M+ SCLC specimens [195]. Additionally, a significant number of transformed tumors with *PIK3CA*-mutations was detected. This frequent occurrence of mutations in *TP53, RB1* and *PIK3CA* is also typical of classic smoking-related SCLC [201] and was reported in previous cases of LACs transforming to SCLC following TKI-therapy, though the inactivation of the p53- and Rb1-signaling pathways more rarely may take place via other genetic/epigenetic mechanisms [68,192,193]. In any case, according to the branching evolutionary path of *EGFR*M+ LAC transforming to SCLC described by Lee et al. [200], the TKI-resistant SCLC clones emerged earlier and at much higher frequency from a founder LAC with complete (homozygous) inactivation of the tumor suppressor genes *RB1* and *TP53* at baseline as compared to LACs with intact p53 and Rb1 function. Indeed, in the former cases the clonal branching of SCLC cells from LAC could be detected even before the TKI-start and the risk of SCLC-transformation was increased >40 times [200].

Taken together the above-mentioned results provide evidence for *TP53* and *RB1* inactivation as predisposing factor for SCLC-transformation of *EGFR*M+ LACs and suggest that evaluating the mutational status of *TP53* and *RB1* at baseline might aid in foreseeing which LACs are more prone to SCLC-transformation following EGFR-TKI therapy [195,197,200]. It remains to be clarified, though, how the presence of *TP53-* and *RB1-*mutations in *EGFR*M+ LACs correlates with the variable time to transformation observed by Marcoux et al. in their patients. Indeed, these authors found a time to transformation from the initial advanced LAC diagnosis ranging from 2 to 60 months (median = 17.8 months) and from TKI-start varying between 1.3 and 53.4 months (median = 15.8 months) [195]. The fact that in certain cases the time to transformation is of several months suggests that additional genetic/epigenetic changes may be required for the phenotypic change to be discernible [198,202]. Conversely, in other patients, tumor progression in association with the LAC-to-SCLC transformation is observed just a few weeks after initiating EGFR-TKIs [68,195] and SCLC clones are detectable before TKI-treatment in LACs with TP53 and RB1 inactivation, thereby justifying the inclusion of this phenotypic change among the possible mechanisms of intrinsic TKI-resistance. Nine percent of the transformed cases in the North American cohort also displayed *EGFR*-amplification, in addition to the founder *EGFR*-mutation [195], implicating that not only EGFR-downregulation but also -upregulation may contribute to the loss of sensitivity to EGFR-TKIs in the SCLC-transformed cells. Although the mechanisms by which SCLC-transformation leads to TKI-resistance need to be addressed more specifically, it is also fair to speculate that the *TP53-, RB1-,* and *PIK3CA-*mutations identified in the transformed tumors may contribute to TKI-resistance, given that these genes regulate a multitude of mechanisms implicated in cell proliferation and survival downstream EGFR.

After transformation, the cohort of Marcoux et al. was treated with platinum-etoposide showing a RR of 54% and a median PFS of 3.4 months, and thereafter with taxanes with a remarkable RR of 50% and median PFS of 2.7 months [195]. This confirmed that adopting the platinum-etoposide protocol used as SOC treatment for conventional SCLC may also be a valid therapeutic choice after the LAC-to-SCLC transformation and that taxanes may represent an interesting alternative for this group of patients, also as late line of treatment. It remains to be clarified which cells are sensitive to and responsible for the significant RR of platinum-etoposide and taxanes in the transformed tumors (i.e., residual responsive LAC cells in transformed tumors or specific sensitivity of the SLC-transformed cells or both?). The SCLC-transformed tumors also exhibited high rate of CNS metastases and median OS since initial diagnosis of advanced lung cancer and after SCLC-transformation of 31.5 and 10.9 months, respectively, which together with the frequent but transient responses to platinum-etoposide are clinical features reminiscent of those in classic smoking-associated SCLC with wt *EGFR* [195]. On the other hand, the short median PFS and OS after transformation indicate that more efficient therapeutic protocols are needed after diagnosing this phenotypic change in TKI-treated *EGFR*M+ LACs. In this regard, the transformed tumors are not always completely insensitive to EGFR-TKIs, as 52% of patients in the North American cohort received TKI-therapy after transformation, mostly in combination with or after cytotoxic chemotherapy, and a few cases showed clinical benefit from this treatment [195]. As in previous reports, this was ascribed to the reemergence of LAC clones in progressing sites after SCLC development [68,195]. In contrast, treatment with immune checkpoint inhibitors yielded no clinical response, resembling the lack of efficacy of immunotherapy in the general population of *EGFR*M+ LAC [203,204]. Notably, a literature review of 39 TKI-treated SLC-transformed LACs (37 *EGFR*M+ cases, 2 *ALK*-positive cases) [198] and a retrospective European cohort of 48 SCLC-transformed *EGFR*M+ LACs [199] displayed time to transformation, RR to platinum-etoposide, and OS since LAC diagnosis or after transformation comparable to those in the study by Marcoux et al. [195,198,199], thereby validating the conclusions in terms of clinical behavior of these tumors.

The above-described studies also underline the relevance of tumor re-biopsies at progression for the histological identification of phenotypic changes such as SCLC-transformation that, as yet, are not detectable in liquid biopsies. Finally, they imply that TKIs may function as factors promoting the SCLC-transformation, especially in NSCLCs with inactivated *TP53* and *RB1*, despite not being essential for the phenotypic transition. In connection with that, the role of *EGFR*-mutations in SCLC-transformation also needs to be elucidated, considering that these mutations are early clonal events involved in the initiation of EGFR-driven LAC [7,11]. In this regard, SCLC-transformation has occasionally been reported in *EGFR*-wt LAC and in LACs driven by *ALK-*rearrangement rather than mutated *EGFR* [198,199], suggesting that *EGFR*-mutations may predispose rather than induce the transformation. Accordingly, some evidence for SCLC-transformation occurring more rapidly in *EGFR*M+ than in *EGFR*-wt LACs has been provided, though after transformation survival and response to platinum-etoposide appear similar in the two groups and resemble those in conventional SCLC [199]. Finally, future multigene analyses will hopefully uncover whether specific genetic signatures of *EGFR*M+ LACs are associated with SCLC-transformation, so that this event can be better predicted and possibly therapeutically counteracted [197]. Most of the reported cases of SCLC-transformation in *EGFR*M+ LAC were treated with TKIs of early generation, while only single patients received osimertinib as first-line [195,198,199]. In addition, recent investigations indicate that in addition to tertiary EGFR-mutations and loss of T790M (“loss of target”), resistance to second-line osimertinib is related to several EGFR-independent mechanisms [205]. Thus, it will be interesting to prospectively analyze how the employment of first-line therapy with osimertinib will impact on the occurrence of SCLC-transformation or other phenotypic changes in patients with *EGFR*M+ NSCLC, since in this group of patients TKI-resistance due to T790M mutation will lose significance.

#### 2.3.2. EMT, BIM Expression, Hypoxia

EMT was initially reported in connection with cases of acquired resistance to EGFR-TKIs of 1G or 2G (<2%) and is now being observed at an increased frequency after the implementation of osimertinib [29]. EMT is characterized by loss of epithelial markers (e.g., the cell-adhesion protein E-cadherin) and acquisition of mesenchymal features, such as spindle-shaped vimentin-positive cells with increased motility, invasiveness, and TKI-resistance. As for the SCLC-transformation, the possibility of NSCLCs containing sarcomatoid spindle cells that have undergone EMT and are intrinsically resistant to EGFR-TKIs prior to treatment cannot be omitted. Alternatively, EMT might be induced very rapidly in some tumor cells after initiation of TKI-treatment as a form of adaptive response to the inhibition of EGFR signaling [206]. Supporting both concepts, anecdotal cases of EMT occurring within weeks of TKI treatment have been reported [206]. Indeed, the transcription factors (TFs) Twist, Snail, Slug and ZEB1, which regulate a plethora of genes associated with a mesenchymal cellular phenotype, can be found upregulated in NSCLC cells before therapy or are rapidly induced by EGFR-TKIs as part of the adaptive cellular reprogramming. In either case, they may induce EMT in NSCLC and lead to resistance to EGFR-TKIs of all three generations [29,206]. Experiments in NSCLC cell lines showed that counteracting EMT can re-establish sensitivity to EGFR-TKIs [207]. However, how EMT causes TKI-resistance remains uncertain. A key event in EMT appears to be the downregulation of the EGFR-interacting adhesion-protein E-cadherin, which is at least in part mediated by epigenetic mechanisms. Indeed, overexpression of the EMT-related zinc-finger transcriptional repressor ZEB1 in *EGFR*M+ NSCLC cell lines inhibits the expression of E-cadherin by recruiting histone deacetylases (HDACs), and this renders these cells insensitive to EGFR-TKIs [207]. Moreover, gene promoter methylation is also involved in E-cadherin downregulation when NSCLC undergoes EMT [208]. Additionally, cases of *ALK*-rearranged NSCLC resistant to the 2G ALK-TKI ceritinib displaying features such as spindled cell shape, loss of E-cadherin immunostaining, and Vimentin overexpression, consistent with EMT, have been documented [209]. Similarly, mutations in genes regulating EMT and E-cadherin expression levels have been reported in crizotinib-resistant *ALK*-positive NSCLCs [210]. Thus, a loss of E-cadherin expression in NSCLC appears to be predictive of poor responsiveness to EGFR- and ALK-TKIs and is characteristic of EMT induction in NSCLCs that become resistant to these drugs.

It has also been shown that the above mentioned, EMT-related TFs can inhibit the transcription of the *BCL2L11* gene. The latter encodes BCL2-like 11 (BIM), a BH3 domain-containing, pro-apoptotic member of the Bcl-2 protein family that is destabilized and downregulated by EGFR-dependent signaling in cancer cells that are EGFR-addicted for survival. Consequently, BIM is stabilized by EGFR-TKIs and thereby contributes in a major way to TKI-induced apoptosis in *EGFR*M+ NSCLC cells [211,212,213,214]. Thus, EMT may induce a TKI-resistant status at least in part via transcriptional suppression of BIM-mediated apoptosis. An additional player contributing to the induction of EMT and EGFR-TKI resistance in NSCLC cells is the teratocarcinoma-derived growth factor 1 (TDGF1)/CRIPTO1, an oncofetal, membrane-associated protein of the EGF-CFC family. *EGFR*M+ NSCLCs intrinsically resistant to EGFR-TKIs were reported to have upregulated expression of CRIPTO1. Moreover, ectopic expression of CRIPTO1 in *EGFR*M+ NSCLC cell lines upregulated ZEB1 and activated the SRC pathway via microRNA-205 (miR-205) downregulation, thereby promoting EMT and erlotinib-resistance of these cells [215]. Conversely, CRIPTO1-overexpressing primary *EGFR*M+ NSCLC cells that were intrinsically erlotinib-resistant became TKI-sensitive upon silencing of CRIPTO1 expression [215]. Intriguingly, miR-205 and the microRNA-200 family are known to repress the expression of ZEB1/ZEB2 and SRC, and in this way can prevent EMT and drug resistance [216,217]. Consequently, ectopic miR-205 overexpression suppressed CRIPTO1-dependent ZEB1 and SRC activation, restoring erlotinib sensitivity in *EGFR*M+ NSCLC cell lines [215]. Also, pharmacologically co-targeting EGFR and SRC synergistically reduced the growth of CRIPTO1-positive, erlotinib-resistant, *EGFR*M+ NSCLC cells, suggesting that this combination might be able to counteract intrinsic resistance to EGFR-TKIs in patients with CRIPTO1-positive, *EGFR*M+ NSCLC undergoing EMT [215].

Interestingly, an intronic deletion polymorphism of the *BCL2L11* gene that results in alternative BIM mRNA splicing and elimination of the pro-apoptotic BH3-domain occurs naturally in a significant fraction of East Asian individuals, with frequency reportedly ranging between 12% and 21% [218,219]. Consequently, this polymorphism impairs the generation of the proapoptotic isoform of BIM required for EGFR-TKI-induced apoptosis and confers an intrinsically TKI-resistant phenotype that can partly explain the heterogeneity of TKI responses across individuals [220]. Indeed, Asian patients with *EGFR*M+ NSCLC, who harbored this host BIM deletion polymorphism, exhibited significantly inferior responses to treatment with TKIs of all three generations and much shorter PFS than individuals lacking the polymorphism, suggesting that the BIM polymorphism is a negative predictive marker of response to EGFR-TKIs [218,219,220,221,222]. Of note, preclinical experiments indicate that BH3-mimetics or HDAC-inhibitors, such as vorinostat, can restore BIM functionality and sensitivity to EGFR-TKIs in *EGFR*M+ NSCLC cells carrying the BIM polymorphism [221,222,223]. In addition to polymorphism, low BIM expression levels in *EGFR*M+ NSCLC samples may also predict poorer initial response and shorter duration of clinical benefit from EGFR-TKIs [57,212,224,225]. The differences in baseline BIM expression levels among NSCLC cases likely reflects heterogeneity within the cellular apoptotic machinery, though what causes these differences remains unclear [57]. Recently, *EGFR*M+ NSCLC patients with low expression level of the transcriptional BIM-inducer Human antigen R (HuR) were reported to display reduced BIM expression, intrinsic resistance to EGFR-TKIs, and significantly shortened PFS, while ectopic overexpression of HuR was able to enhance sensitivity to gefitinib in NSCLC cells in vitro and in vivo [226].

The TAM (Tyro3, AXL, MerTK) family of RTKs has oncogenic potential and both the expression of MerTK and AXL can increase in *EGFR*M+ NSCLC treated with EGFR-TKIs and induce acquired resistance to these drugs [227]. MerTK functions as by-pass track and activates MAPK- and FAK-signaling, thereby converging downstream EGFR, while AXL-signaling has been associated with acquired resistance through the induction of EMT [227,228]. Some evidence for pre-existing, drug-tolerant cell clones overexpressing AXL at baseline has been recently presented in single cases of *ALK*-rearranged NSCLC not responding to crizotinib [229]. Thus, it would be relevant to investigate in biopsies obtained before treatment and early during response to therapy whether populations of AXL-overexpressing cells exist in NSCLC at baseline, as a source of rapid EMT development and primary TKI-resistance shortly after therapy initiation. This approach would also allow clinical validation of the alternative possibility emerged from studies in TKI-treated NSCLC cell lines that AXL and EMT are promptly induced as part of the rapid reprogramming these cells go through after TKI-initiation. It is postulated that this adaptive response results in de-repression of certain alternative RTK-mediated by-pass pathways that ultimately allow some cells to survive the treatment, proliferate, and even switch to a more mesenchymal, less EGFR-dependent phenotype, thereby persisting as a form of “residual disease” [206].

Cancer-associated fibroblasts (CAFs) have been implicated in the induction, through paracrine mechanisms, of EMT and TKI-resistance in NSCLC. For instance, by culturing *EGFR*M+ NSCLC cell lines with CAFs isolated from NSCLC tissues, Yi et al. were able to promote EMT and EGFR-TKI resistance of the cancer cells. This was at least in part due to the secretion of HGF and insulin-like growth factor-1 (IGF-1) by the CAFs that activated signaling pathways in the NSCLC cells leading to EMT and TKI-resistance [230].

An additional factor related to the cancer microenvironment that can induce resistance to EGFR-TKIs is hypoxia, which is present to a variable extent in metabolically active solid tumors such as NSCLC. Acute hypoxia due to repetitive transient vessel occlusion by the growing tumor mass and chronic hypoxia caused by limited diffusion of nutrients and oxygen in tumor cells distant from vessels dynamically coexist in cancers. While a persistent hypoxic state can gradually reduce the efficacy of TKIs and result in acquired resistance to these drugs, a rapidly induced hypoxic microenvironment may also contribute to intrinsic TKI-resistance. Through activation of the hypoxia-inducible transcription factors HIF-1α and HIF-2α, hypoxia regulates a plethora of downstream genes and promotes genomic instability, tumor tissue acidosis, angiogenesis, invasiveness, and tumor progression associated with increased metastatic behavior, which ultimately result in poor prognosis and resistance to apoptosis-mediated cancer therapies such as radio-chemotherapy, targeted therapy, and immunotherapy [231]. Uncontrolled EGFR-signaling is associated to hypoxia, while preclinical and clinical investigations have shown that EGFR-TKIs can increase the hematic flow through the tumor tissue and reduce hypoxia [231,232]. On the other hand, hypoxia renders *EGFR*M+ NSCLC cell lines TKI-resistant through activation of insulin-like growth factor 1 receptor (IGF1R), a known negative prognostic marker in advanced NSCLC [233,234]. In turn the aberrant IGF1R-signaling favors the generation of primitive cancer stem cells and EMT, whereas IGF1R-inhibitors can revert these phenomena, thus, targeting IGF1R may have the potential to prevent hypoxia-induced cancer progression, EMT and ultimately TKI-resistance [234,235,236]. Consequently, the combination of EGFR-TKIs with hypoxia-targeted therapies is currently being investigated preclinically and clinically with the purpose of improving the effect of EGFR-TKIS [231].

#### 2.3.3. Conversion to SqCC

In addition to transformation to SCLC and EMT, there is mounting evidence for the association of TKI-resistance with another phenotypic change, namely the transition of an *EGFR*M+ LAC to a SqCC during TKI-treatment. According to the 2015 WHO classification of lung tumors, adenosquamous carcinomas (defined as carcinomas where the adenomatous and squamous components represent each at least 10% of the whole tumor tissue) account for no more than 4% of all lung cancers [237]. However more recent studies showed that up to 10% of NSCLCs may contain mixed adenomatous and squamous areas in the same primary tumor. Regardless of their size and prevalence, these components frequently share identical oncogenic alterations in cancer-drivers such as mutations in *EGFR, KRAS, AKT1, ERBB2,* and *PI3KCA* genes or fusions of *ALK* and *RET* genes, with frequencies resembling those in pure LAC, thereby suggesting a potential phenotypic transition [7,238]. Indeed, the trans-differentiation from LAC to SqCC has been described both in humans and in mouse models, often when tumor cells are characterized by inactivation of the tumor suppressor gene *LKB1/STK11*, which occurs in up to 20% of LACs [3,7,10,238,239]. Moreover, clinical investigations have identified cases revealing a phenotypic LAC-to-SqCC change at progression during treatment with EGFR-TKIs of all three generations [30,240], which is consistent with the association of this phenotypic conversion with TKI-resistance [7,238]. However, as for SCLC-transformation and EMT, it is debated whether the conversion from LAC to SqCC is a clonal selection or an adaptive histological change resulting in phenotype-switch [240]. Thus, it cannot be excluded that a certain amount of tumor cells with SqCC phenotype might already be present before the initiation of TKI-treatment and immediately act as mechanism of poor therapeutic response.

A recent pooled analysis of published case reports or small case series of SqCC-transition in *EGFR*M+ LACs included 16 patients treated with 1G or 2G TKIs as first/second/third-line therapy and 1 receiving osimertinib as second-line [240]. As baseline features, the percentage of females (82%), median age (63 years), and percentage of smokers (41%) were higher than in the general population of *EGFR*M+ LAC patients [1,2,90]. The founder *EGFR*-mutations in baseline LAC samples were exon 19dels (all E746_A750del) and L858R in 53% (9/17) and 41% (7/17) of cases, respectively, thereby resembling the mutation distribution in the general population of patients with *EGFR*M+ LAC [1,2,90]. The remaining case (6%) harbored a de novo T790M mutation. As observed in most cases transforming to SCLC, all the 17 SqCC-converted cases maintained the original *EGFR*-mutations [240]. This substantiates the concept that the new SqCC phenotype observed at disease progression originates from a founder LAC [240], considering that <5% of pulmonary SqCCs display activating *EGFR*-mutations [8]. Given that diagnostic biopsies are small and taken from single sites, it cannot be excluded though, that some of the cases described as SqCC-conversion of *EGFR*M+ LACs, in fact were at baseline *EGFR*M+ adenosquamous carcinomas, which are known to harbor *EGFR*-mutations in both components [241] and could have progressed through further clonal selection of the SqCC-population [240]. The available genotyping data from the 11 samples tested after the onset of the SqCC phenotype revealed the emergence of the TKI-resistant mutant T790M in eight cases, PIK3CA-mutation in two cases, and the occurrence of S768I in one case [240]. The high frequency of T790M in the SqCC-converted specimens contrasts with the low incidence of newly acquired T790M and tendency to lose pre-existing T790M observed in the *EGFR*M+ LACs undergoing SCLC-transformation (see Section 2.3.1) [195,198]. The appearance of T790M in a considerable number of SqCC-converted LACs following TKI-therapy also raises the question whether this mutation is the main mechanism of TKI-resistance in these tumors, rather than other molecular events associated with the squamous phenotype. More molecular profiling of SqCC-converted LACs at baseline and after conversion is needed to define molecular signatures that could predispose to this phenotypic change and/or render it resistant to TKI-treatment.

In terms of clinical outcomes, the pooled literature analysis of Roca et al. [240] displayed a median duration of TKI-treatment prior to SqCC-conversion of 11.5 months (range 4–69 months), thus shorter than the median time from TKI-start to SCLC-transformation reported by Marcoux et al. (15.4 months) [195] and Roca et al. (18 months) [240]. The median OS after diagnosis of NSCLC was of 20 months in the cases experiencing SqCC-conversion, which is shorter than the OS observable in the general population of *EGFR*M+ LAC not undergoing phenotypic changes. It is also shorter than the above-mentioned OS of 31.5 months from diagnosis observed in the *EGFR*M+ LACs undergoing SCLC-transformation [195]. The treatment after the SqCC-transition was described for only 12 patients in the pooled analysis of cases [240] and included chemotherapy (24%), TKI (41%), or a combined protocol (6%). The clinical benefit was quite modest: two patients did not benefit from any therapy and died shortly after very rapid PD, while only four patients exhibited a PR after administration of a 3G EGFR-TKI. After SqCC-conversion the median OS was therefore of only 3.5 months, i.e., significantly worse than that after SCLC-transformation, reportedly ranging between 6 and 10.9 months [195,198,199]. These discrepancies between SqCC-conversion and SCLC-transformation after treatment with EGFR-TKIs suggest that the former phenotypic event may be associated with worse prognosis, however larger series need to be investigated and corrected for potential biases, such as the smoking habit, before reaching firmer conclusions. Indeed, the cohort of SqCC-converted LACs comprised more smokers than the reported cohorts of LACs transforming to SCLC [195,198,240], which could be a bias or a factor contributing to the transition to the SqCC phenotype.

As for the SCLC-transformation and EMT, there is still a lack of markers capable of revealing the phenotypic change from LAC to SqCC in plasma samples during treatment with TKIs [7,242]. Therefore, the recognition of SqCC-conversion, as for the other phenotypic changes, relies on histological and immunohistochemical investigations performed on biopsies from recurrent/progressing sites. However, these can be challenging for pathologists. For instance, given the small sizes of these biopsies and the prominent morphological intra- and inter-lesion heterogeneity of advanced NSCLC, these phenotypic changes may not necessarily be represented in the examined tissue samples and therefore can be missed in certain patients that are resistant to EGFR-TKIs. Additionally, because of clonal heterogeneity, both genetic and phenotypic changes associated with TKI-resistance in advanced NSCLC might be present only in some, but not all the progressing lesions [7,11,68]. Although this issue can be addressed by taking biopsies from more than one site, the invasiveness and side effects related to this approach renders the expected future possibilities of detecting biomarkers for phenotypic changes in liquid biopsies or by molecular imaging particularly attractive [243,244,245].

### 2.4. Autophagy, Drug Efflux or Sequestration

Although it has only been studied in preclinical models based on cell lines and tumor xenografts in mice, autophagy activation is potentially considerable among the mechanisms of resistance to TKIs, and combined therapy with TKIs and autophagy inhibitors appears as a promising approach to augment the possibility of eliminating RTK-dependent tumor cells [246,247,248]. One of the effects of TKIs is to reduce the activity of the PI3K/AKT/mTOR pathway and conceivably this may result in rapid autophagy induction, given that among other functions, this signaling pathway normally blocks autophagy initiation. Once derepressed by TKIs, autophagy proceeds to formation of autophagolysosomes, which can degrade their content and release primary cellular components in the cytosol for recycling and reuse. In stressful situations this process functions to let the cells recover in standby until cellular homeostasis is re-established. In this respect, it has been shown that cell lines representing different EGFR-addicted cancer types, including NSCLC, may under therapeutic stress by TKIs use autophagy to eliminate the drugs and survive the treatment, while inhibitors of autophagy may augment the growth inhibitory effect of EGFR-TKIs [246,247,248,249]. Although not necessarily active in cancer cells before treatment, the rapid induction of autophagy by TKIs can operate as prompt negative feedback-mechanism reducing drug efficacy and leading to rapidly acquired resistance. However, in cancer cells with pre-existing autophagic activity the further boost of autophagy by TKIs could result in immediate lack of therapeutic response, thereby representing a form of primary TKI-resistance.

Thus, blocking autophagy might represent a future strategy to overcome intrinsic/acquired resistance and increase sensitivity to EGFR-TKIs in *EGFR*M+ LAC. Still, much more work is needed to elucidate the potential role of autophagy in TKI-resistance and the possibility of enhancing the efficacy of TKIs of all generations. Somewhat conflicting data were recently published on this issue. One report suggested that one of the mechanisms by which osimertinib inhibits the growth of *EGFR*M+ NSCLC cells in vitro and in mouse xenografts was by triggering autophagy-mediated cell death [250]. Another just released report did confirm that osimertinib, like the EGFR-TKIs of 1G and 2G [246,247,248,249], increases the autophagic activity of *EGFR*M+ NSCLC cells in vitro and in xenograft mice, however, it concluded that this resulted in the development of osimertinib-resistant cells exhibiting stem cell-like properties [251]. Thus, as previous results have suggested, autophagy may be a double-edge sword capable of either promoting or restraining the survival of *EGFR*M+ NSCLC cells in hypoxic situations and the EGFR signaling may be a key modulator of the switch between cancer cell survival and death in these conditions [252,253]. Thus, even if autophagy may potentially participate to the elimination of cancer cells, hypoxic NSCLC regions may also contribute to tumor progression due to the ability of *EGFR*M+ NSCLC cells to adapt to the adverse conditions, at least in part, through autophagy [252,253]. Summing up these results, it is fair to conclude that more work is warranted to elucidate whether and how autophagy may contribute to acquired and intrinsic TKI-resistance in the clinical setting.

Resistance to EGFR-TKIs may also be due to increased drug-efflux mediated by ATP-binding cassette transporters residing in the cell membrane of NSCLC cells that can pump these drugs out into the extracellular environment [254]. Alternatively, TKIs may be sequestered in lysosomes, protonated, and subsequently removed from cancer cells by exocytosis or via the efflux transporters, thereby precluding the interaction of TKIs with EGFR [254]. Initial observations indicated that being the EGFR-TKIs substrates of ATP-binding cassette transporters, such as P-glycoprotein (Pgp), they could be utilized as a synergistic strategy for antagonizing Pgp-mediated resistance to chemotherapeutic drugs in NSCLC cells not harboring sensitizing *EGFR*-mutations [255,256]. Yet, the induction of specific drug efflux transporter proteins, including Pgp, that may occur in *EGFR*M+ NSCLCs treated with EGFR-TKIs is a mechanism that reduces the intracellular TKI concentration and contributes to acquired resistance to these drugs [254]. It remains to be established to which extent multidrug-resistance transporter proteins and lysosomal trapping may operate as mechanisms of intrinsic TKI-resistance in NSCLC cells.

### 2.5. MicroRNAs and Long Non-Coding RNAs

The role of non-coding RNAs, such as microRNAs (miRs) and long non-coding RNAs (lncRNAs) in acquired and intrinsic resistance to EGFR-TKIs is a vast subject of intense preclinical investigation, which is beyond the scope of this review. The results have hitherto been achieved primarily using cell lines and their clinical impact remains to be clarified. Here is worth just mentioning that a multitude of miRs and lncRNAs, as in other cancer types, appear down-regulated in NSCLC, including several EGFR-targeting miRs (miR-7, miR-27a-3p, miR-30, miR-34, miR-128, miR-133, miR-134, miR-145, miR-146, miR-149, miR-218, and miR-542-5p) [257] and lcnRNAs [258,259]. Reestablishing the expression of miRs using miRNA-mimics delivered via viral and non-viral (locked nucleic acids or liposomal nanoparticles) systems into the tumor tissue of NSCLC patients might provide an additional tool for suppressing tumor growth and improve the efficacy of EGFR-TKIS. However, safety and efficiency concerns related to the potential immunogenicity, mutagenicity, and toxicity induced by viral vectors or the low miR-delivery rate and off-target effects of non-viral vectors are still limiting the clinical application of these strategies [257].

Among miRs that instead are upregulated in NSCLC, miR-21 is particularly interesting, as it is frequently overexpressed in *EGFR*M+ NSCLC cell lines, in which it contributes to TKI-resistance by downregulating Phosphatase and TEnsin homolog (PTEN) and Programmed Cell Death protein 4 (PDCD4) expression and thereby activating the PI3K-AKT pathway [260,261]. Moreover, inhibiting miR-21 expression promotes apoptosis in these cells and suppresses tumor growth in nude mice treated with EGFR-TKIs. Consistent with that, miR-21 overexpression was detected in the tumor tissue of NSCLC patients that became resistant to EGFR-TKIs and correlated with poor response and shorter OS after this therapy. By the same token, miR-21 expression in the plasma of EGFR-TKI-treated NSCLC patients was higher at the time of acquiring resistance than at baseline [260,261]. Furthermore, large-scale miR-profiling of serum samples from NSCLC patients treated with EGFR-TKIs showed that among 153 differentially expressed microRNAs, miR-21, AmiR-27a, and miR-218 were significantly upregulated in patients showing no response to these drugs as compared to sensitive patients, suggesting that these three microRNAs may also be implicated in intrinsic resistance to EGFR-TKIs [262]. Finally, as mentioned in Section 2.2.3, preclinical models have shown that aberrant MET signaling due to MET-amplification renders *EGFR*M+ NSCLC cells TKI-resistant by promoting their proliferation and survival via reactivation of the MAPK and PI3K-AKT pathways that are inhibited by EGFR-TKIs [138]. This in part caused by MET upregulating the expression of miR-21, miR-30b/c, and miR-221/222, which in turn represses the tumor suppressor PTEN and the proapoptotic effectors BIM and APAF-1 [138]. MET also downregulates miR-103 and miR-203, which are negative regulators of EMT, thereby contributing to EMT-mediated TKI-resistance [138].

The mechanisms by which altered expression of lncRNAs affect the expression of gene products that may contribute to drug resistance, including TKI-resistance, remains poorly known [263]. Certain lcnRNAs that are overexpressed in NSCLC, such as MIR31HG and UCA1, are capable of interfering with the response to EGFR-TKIs by activating the PI3K/AKT/mTOR pathway [263,264]. Moreover, UCA1 and other overexpressed lncRNAs, such as MALAT1/NEAT2 and BC087858, appear to be involved in EGFR-TKI resistance by modulating the EMT [263]. Downregulation of lcnRNAs targeting the EGFR- and IGF1R-signalling, such as GAS5, has also been implicated in TKI-resistance [258]. Future studies need to further clarify how and when lcnRNAs are involved in primary and secondary resistance to EGFR-TKIs in NSCLC patients.

## 3. Further Considerations Regarding the 3G EGFR-TKI Osimertinib

The literature on inherent resistance to 3G EGFR-TKIs primarily concerns osimertinib and is limited, given that this drug is approved as second line for T790M-positive, *EGFR*-mutant NSCLC patients, who have progressed on 1G/2G EGFR-TKIs. Once osimertinib becomes the new SOC for first-line therapy with EGFR-TKI, as recent data strongly advocate for [24,25], it will be easier to reveal and understand the potential causal mechanisms of intrinsic resistance to this drug. As mentioned above, several altered signaling pathways leading to acquired resistance to osimertinib have been discovered and 5% to 15% of T790M-positive patients have reportedly shown inherent resistance to this drug [25,29,31,32,33,35,36,37]. In addition, the rate of CR in responders to second line osimertinib is low. This is often due to remaining lesions that are a source of new progression in most patients and that may be T790M-negative and/or possess other mechanisms of TKI-resistance [24,25,29,31,33,35,36,37]. The acquisition of tertiary mutations within the *EGFR*-gene, such as C797S that impairs the covalent binding between the cysteine residue at position 797 of EGFR and osimertinib, is specifically induced by osimertinib treatment. In contrast, *EGFR*-amplification and the above-described EGFR-independent resistance mechanisms are shared by EGFR-TKIs of all three generations. This also means that if an *EGFR*M+ NSCLC becomes resistant to a first-line TKI of 1G/2G through one or several of these shared mechanisms, it will be intrinsically resistant to osimertinib. Accordingly, there are reports of cases not responding to osimertinib or rociletinib, which showed *EGFR-, ERBB2-* or *MET-*amplification, or SCLC transformation in samples obtained before or after very few weeks of treatment [29,242]. Similarly, as mentioned in the Introduction, *RTK*- or *BRAF*-fusions or *KRAS*-mutations concomitant with the loss of the T790M mutation and preservation of the original activating *EGFR*-mutant have been identified in cases exhibiting very rapid progression (temporally consistent with intrinsic resistance) and poor survival on second-line osimertinib [32,36,37].

Additionally, Blakely et al. [12] analyzed the mutational profile of cfDNA isolated before osimertinib-treatment from a group of 20 *EGFR*M+ NSCLC patients responding to subsequent administration of osimertinib and from 21 non-responders. They detected co-alterations in *MET* (3/21), *NF1* (5/21), *CDK4/6* (3/21), *CCNE* (3/21), *PIK3CA* (6/21) and *APC* (5/21) only in the non-responders and found that alterations in cell cycle genes such as *CDK4/6* or genes of the MAPK-/PI3K-/WNT-pathways were associated with lack of response to osimertinib and shorter PFS. These results emphasize that genetic co-alterations of these pathways may play an important role in intrinsic resistance to osimertinib treatment and could be employed as negative predictors of response to this drug [12]. Finally, recent data point to the up-regulation of AXL as alternative mechanism of intrinsic resistance to osimertinib. Gene expression analysis in two cohorts of *EGFR*M+ NSCLC patients treated with gefitinib or osimertinib showed frequent concomitant overexpression of AXL and CUB domain-containing protein-1 (CDCP1) in cases with poor response to these drugs [265]. Another study in NSCLC patients treated with 1G/2G TKIs or osimertinib found an association between high AXL expression and low RR as well as early tumor progression [266]. This study also showed that treating cultured *EGFR*M+ NSCLC cells with osimertinib resulted in AXL upregulation as well as interaction with EGFR and ERBB3, which in turn contributed to maintaining cell survival and to the emergence of osimertinib-tolerant cells. Cell-based assays showed that adding AXL inhibitors in tumor cell- or patient-derived xenograft models synergized with osimertinib by reducing the viability of osimertinib-treated NSCLC cells. This hindered the emergence of osimertinib-tolerant cells, thereby delaying tumor recurrence in the subcutaneously grafted mice [266]. These results suggest that the AXL signaling plays an important role in *EGFR*M+ NSCLC cells regarding both the initial adaptive response and intrinsic resistance to osimertinib as well as the development of long-term drug-tolerance. Thus, AXL overexpression might be used as a novel biomarker for the initial tolerance and intrinsic resistance of *EGFR*M+ NSCLCs to EGFR-TKIs, but this possibility necessitates further validation in larger studies.

An additional consideration from the above-mentioned results is that when the occurrence of T790M in patients progressing on early generation EGFR-TKIs is determined only by mutation analysis of plasma cfDNA before allocation to osimertinib treatment, one may risk missing a possible concomitant transformation to SCLC or SqCC, as well as cases with EMT or AXL overexpression, thereby neglecting these causes of primary resistance to osimertinib [72,242]. Finally, among the so-far-identified causes of primary osimertinib-resistance, a recently reported case with de novo occurrence of the rare *EGFR* L747P mutation in exon 19, should be mentioned (see Section 2.1). This mutation conferred lack of response and intrinsic resistance to both gefitinib and osimertinib [99]. Further cases not responding to and rapidly progressing on first-/second-line osimertinib need to be molecularly investigated for properly understanding and validating the mechanisms of primary resistance to this drug.

Notably, regardless of the resistance-mechanism involved, most osimertinib-resistant cases maintain the original activating *EGFR-*mutation even if they lose T790M, suggesting that EGFR continues to be an essential driver in the resistant cells and justifying the implementation of combinatorial therapeutic strategies aimed at re-sensitizing them to osimertinib [29,32,33,35,36]. In this respect, there is emerging indication that both the presence and the relative concentration of T790M may impact the initial response to osimertinib and possibly other 3G TKIs. Indeed, in the phase I/II AURA trial for patients with advanced NSCLC progressing during treatment with 1G/2G EGFR-TKIs, the median PFS on osimertinib was 9.6 and 2.8 months in T790M-positive and -negative cases, respectively [93]. An analogous phase I/II study in which patients progressing on 1G/2G TKIs received the other 3G TKI rociletinib showed an objective RR of 59% for the evaluable T790M-positive cases and 29% for the T790M-negative ones [267], confirming that the presence of T790M predicts better response to 3G EGFR-TKIs. Moreover, NSCLC patients with a high T790M/activating *EGFR*-mutation ratio in tumor samples or in plasma cfDNA have displayed a significantly better RR to second-line osimertinib and a longer PFS than patients with a low ratio [268,269]. Comparably, in a retrospective study Li et al. recently observed that quantitative measurements of T790M mutant copy number in plasma cfDNA by digital droplet PCR (ddPCR) may predict treatment response and outcome after osimertinib in NSCLC patients resistant to 1G/2G TKIs [270]. In this cohort, patients exhibiting PR or SD to second-line osimertinib had higher T790M mutant copy number in cfDNA than those with PD. In addition, a high T790M copy number (≥105 copies/mL of plasma) was associated with longer PFS and OS [270]. However, in another *EGFR*M+ cohort receiving second-line osimertinib after identifying T790M in cfDNA, patients with a high T790M copy number (≥10 copies/mL) showed a (non-significant) trend of shorter PFS and OS compared to those with a low T790M copy number (<10 copies/mL) [271]. Thus, additional studies are needed to clarify the predictive value of different quantitative measurements of T790M abundance for osimertinib-treatment in NSCLC. In particular, the predictive suitability and the best cut-off values of the T790M/activating *EGFR*-mutation ratio, T790M relative AF, and T790M concentration in different types of specimens ought to be further validated, compared, and optimized before clinical implementation not least because these parameters may also be influenced by different biological aspects (for example amplification of T790M-positive *EGFR*) [61].

It has also been observed that when C797S develops in NSCLCs that do not carry T790M and are treated with osimertinib in the first-line setting, these tumors become resistant to 3G TKIs but may remain responsive to 1G TKIs [272,273]. Combination therapy with osimertinib and a 1G EGFR-TKI appears effective in these cases if tumors harbor C797S and T790M located in trans allelic configuration that allows targeting of both mutants [273,274]. However, it has been noticed that after an initial OR, further progressive disease may occur due to tumor cell clones shifting C797S from in trans to in cis with respect to T790M, which results in steric hindrance of the binding of TKIs to [274]. An additional factor influencing the response to 3G TKIs appears to be the presence, amount, and type of the co-existing original sensitizing *EGFR*-mutations. A recent Taiwanese study showed that among patients treated with second-line osimertinib after progressing during 1G/2G EGFR-TKI treatment because of the appearance of a T790M mutation, those without detectable original *EGFR*-activating mutations in plasma before osimertinib initiation had the best median OS and PFS (22.4 and 10.8 months, respectively). In contrast, patients without detectable T790M but presence of *EGFR*-activating mutations in their cfDNA samples displayed the shortest median PFS in the cohort (2.6 months) [30]. Similarly, in the above-mentioned study by Del Re et al. the PFS after receiving second-line osimertinib was significantly shorter in patients with high AF of the sensitizing *EGFR*-mutant in their cfDNA than in patients with low AF [269]. This is consistent with the fact that the abundance of T790M and co-existing activating *EGFR*-mutation inversely affect the predictive impact of the T790M/activating *EGFR*-mutation ratio.

In addition, in vitro testing of *EGFR*-mutants capable of conferring osimertinib-resistance regardless of the presence of T790M (therefore, also when used as first-line) showed that when exon 19del was the sensitizing mutation, only C797S imparted significant resistance against osimertinib. In contrast, either of the combinations of L858R with C797S, C797G, L718Q, or L718V mutations conferred resistance to osimertinib, indicating that the type of co-existing sensitizing *EGFR*-mutation may affect the resistance to first- or second-line osimertinib too [275]. Similar results have recently been seen in T790M-positive NSCLC patients receiving osimertinib as second- or third-line, in that those with co-existing *EGFR* exon 19del displayed longer PFS and OS than patients harboring L858R co-mutation [23]. Consistent with the results by Niederst et al. [272], erlotinib showed the greatest activity for C797S-mediated resistance, whereas the 2G TKIs afatinib and dacomitinib were effective for other osimertinib-resistant mutations [275]. In line with that, C797S has been observed to develop instead of T790M in subsets of EGFR L858R- and G719A-positive cell lines that became resistant to increasing concentrations of afatinib or dacomitinib. These C797S-harboring cell clones, despite being also osimertinib-resistant, responded to erlotinib or gefitinib, while as expected cells that had acquired T790M were sensitive to osimertinib but not 1G TKIs [276,277]. Together, these results suggest that 1G or 2G EGFR-TKIs might help tackle resistance to osimertinib if this drug is employed as first-line and depending on the combinations of secondary and sensitizing mutations [275]. Additional preclinical results further suggest that 1G TKIs could be more effective than 2G TKIs as second-line treatment to the C797S/sensitizing mutation combination emerging after first-line osimertinib [278]. Interestingly, in a very recent study an initial combination of osimertinib and afatinib appeared capable of eliminating exon 19del-positive cells with no development of T790M and C797S resistance-mutations, while the sequential use of the two drugs was unable to do so and resulted in the growth of triple exon 19del/T790M/C797S mutants [279]. The different combinations of osimertinib with 1G or 2G EGFR-TKIs await clinical testing in specific trials.

These accumulating data also imply that re-biopsies should be performed at the time of progression on first-line EGFR-TKIs of early generation and thoroughly analyzed histologically, by NGS, and other ancillary techniques of PCR, FISH, and IHC for the possible presence of shared molecular and phenotypic resistance-mechanisms, before considering second-line treatment with osimertinib in T790M-positive cases. When feasible, a tumor tissue re-biopsy should be performed together with a liquid re-biopsy, given that cfDNA/RNA from liquid biopsies can be problematic for the detection of potentially occurring gene fusions and cannot assess the presence of SCLC-transformation, EMT or trans-differentiation to SqCC [242]. However, given their high achievability and ability to overcome the problem of genetic tumor heterogeneity, liquid biopsies analyzed by NGS are useful for identifying circulating T790M and possible co-mutations before initiating osimertinib, and for monitoring the response and development of resistance-mutations during treatment [12,29,268,269,270,271].

## 4. Conclusions

Cases of *EGFR*M+ NSCLC with poor response to EGFR-TKIs due to pre-treatment co-mutations in other cancer-drivers have been documented by several groups [12,17,38,44,51,52,68,116,117,118,124,139,140,141]. From what is discussed above, it is increasingly established that once treated with EGFR-TKIs, NSCLCs that are dependent on EGFR-signaling may become TKI-resistant by selecting pre-existing clones carrying resistance-mutations or possessing the ability to depend on alternative oncogenic pathways for growth and survival, even if the initial TKI-sensitive clones are eliminated [11,12,17,29,63,66,139,192]. This reflects tumor heterogeneity at a mutational and chromosomal level [7,11] and the fact that the majority of advanced *EGFR*M+ LACs not only depends on EGFR but also on multiple co-occurring oncogenic events [12]. As mentioned, several of the genetic mechanisms underlying the “acquired” TKI-resistance may already be present at sufficiently high AF at baseline (de novo) or be very rapidly induced in surviving cells as early adaptive tumor response to the targeted therapy. Thereby, these genetic changes may promote the “intrinsic” resistance that typically ensues within the first 3 months after initiating the TKI-treatment [17,29,57,206]. Due to tumor heterogeneity, different random genetic events causing intrinsic and acquired resistance may be concomitantly present within the same tumor or in separate metastatic sites within the same patient (i.e., polyclonal resistance) [12,57]. During further tumor evolution the most effective clones for tumor progression under the adverse conditions caused by targeted treatment may be selected and expanded. Indeed, while sensitizing *EGFR*-mutations are prevalently occurring as early clonal events during NSCLC development, most advanced NSCLCs possess heterogeneous regions harboring late clonal driver alterations that can represent TKI-resistance mechanisms, such as mutations in *TP53* and genes involved in the RAS-RAF-MAPK or PI3K-AKT-PTEN-mTOR pathways, cell cycle regulation, Wnt/β-catenin pathway, DNA damage repair, chromatin remodeling, and histone methylation [11,12].

As an additional layer of complexity, an alternative, recently described mechanism by which cancer cells can be intrinsically or become secondarily resistant to EGFR-TKIs is “drug tolerance”. This is considered an acute defense response preceding a completely drug-resistant state and tumor progression [280]. Across multiple cell lines, small subpopulations of cancer cells have been reported to survive in response to a variety of drug treatments, including targeted therapies, by initially entering a so-called drug-tolerant “persister” state of negligible growth lasting weeks to months during treatment. The increasing interest in drug tolerance to EGFR-TKIs may provide new possibilities for counteracting intrinsic/acquired resistance to these drugs in NSCLC. Ramirez et al. demonstrated in EGFR-addicted, erlotinib-sensitive NSCLC cell lines the emergence of small subpopulations of cancer cells that became resistant to erlotinib and originated from a common clone, through the bottleneck of drug-tolerant, slow-growing “persisters” [280]. Using large-scale drug screening and whole-exome sequencing, these authors provided pharmacological and/or genetic evidence for emergence of diverse alternative mechanisms of drug-resistance in the erlotinib-resistant cell colonies, including ones observed clinically such as T790M or mutations/CNV in alternative parallel oncogenic RTKs (MET or ERBB2) or in the downstream RAS-RAF-MAPK and -PI3K-AKT-PTEN-mTOR pathways. Interestingly, Ramirez et al. identified erlotinib-resistant colonies with multiple concurrent genetic alterations and/or drug vulnerabilities, while they were unable to determine the erlotinib-resistance mechanisms in other drug-resistant colonies, which suggests that the diversity and heterogeneity of TKI-resistant cells emerging from the “persister” state could be even greater than anticipated [280]. Indeed, several questions regarding the timing, diversity and mechanisms by which TKI-resistance can arise in patients through the “persister” bottleneck in different selective pressures remain unanswered.

In this respect, epigenetic mechanisms of reversible drug-tolerance to osimertinib have been described. One study showed that miR-147b can generate a reversible state of tolerance to osimertinib [281]. Using miRNA-sequencing analysis, metabolomics and genetic studies, these authors demonstrated that miR-147b is the most upregulated microRNA in *EGFR*M+ NSCLC cells that are osimertinib-tolerant and that this drug-tolerance, at least in part, is due to the deregulation of the tricarboxylic acid cycle and induction of pseudohypoxia pathways by this miR [281]. Although hypoxia is the main stimulus for the expression of the transcription factors HIF-1α and HIF-2α that regulate downstream hypoxia-driven genes, HIF-1α and HIF-2α can also be induced by a pseudohypoxia state. In turn, this state can be caused by aberrant growth factor signaling and mutations in oncogenes or tumor suppressor genes independently of oxygen levels [282]. The work by Zhang et al. indicates that miR-147b is an additional trigger of pseudohypoxia and thereby can initiate tolerance to EGFR-TKIs in LAC [281].

Many of the studies discussed above indicate that the molecular characterization of inherently TKI-resistant pre-existing clones is ongoing [11,12,17,29,63,66,139,192,193,195,200,240], yet many aspects of their nature remain elusive. For instance, a still open question is whether they consist of more primitive cancer cells resembling cancer-stem cells. In this regard, markers of primordial cancer stem-cells, such as Oct4 and CD133, have been detected in NSCLC cell lines and in tumor specimens of *EGFR*M+ NSCLC patients with acquired resistance to EGFR-TKIs, suggesting that they play a role in the resistance process [283]. In any case, regardless of their resemblance to cancer-stem cells, the fact that the resistant clones implicated in intrinsic resistance, are pre-existing at baseline implicates that they are TKI-independent. However, the clones can further expand into a larger population of TKI-resistant cells under the selective pressure of the following treatment with EGFR-TKIs.

Regardless of which of the above-described cellular paths causes intrinsic TKI-resistance, i.e., through the random pre-existence of clones with resistance-conferring genetic alterations [11,12], or the rapid, adaptive, transcriptional cancer cell reprogramming upon treatment [206], or through the bottleneck of a drug-tolerant, slow-growing “persister state” [280], different and quite heterogeneous drug-resistance mechanisms may co-exist in the same TKI-resistant NSCLC. This situation appears particularly challenging for “personalized” treatment, as even if an effective combination therapy may be used against one TKI-resistant cell subpopulation, it may not necessarily be effective for other subpopulations that possess other resistance-mechanisms and that in practice may have been undetected [280]. In this regard, “persisters” represent such a minor fraction of the bulk cancer population that they are currently difficult to study in a clinical context, and there is no known molecular signature of having passed through the “persister” state clinically [280].

Collectively, the different concepts underlined in this review support the view that intrinsic and acquired resistance to EGFR-TKIs are strictly connected to each other and may differ mainly for the time point in which they can be objectively perceived (immediately/few weeks vs. several months after TKI-initiation). In turn, this temporal difference may depend on the amount and operational potency of the preexisting/early induced resistant tumor cells as well as on the interindividual differences in TKI metabolism and pharmacokinetics. Mutations potentially causing primary TKI-resistance might be difficult to detect in formalin-fixed paraffin-embedded tissue biopsies if they are only present in small heterogeneous subclones and if the DNA sequencing coverage is suboptimal. Conversely, in acquired TKI-resistance, the causative mutations should be easier to identify, as due to treatment-related selective pressure they should be present in most cancer cells at progressing sites. Consequently, targeting a single activating *EGFR*-mutation will eventually result in treatment failure, because pre-existing or swiftly induced resistant cells will, by variable mechanisms and at different times and tumor locations, expand and prevail. By the same token, the combination of drugs targeting alterations in different pathways that are already identifiable at baseline could potentially be utilized to prevent or postpone the appearance of resistant tumor cells more effectively than sequential monotherapies with TKIs of different generation [206]. Indeed, given the increasing evidence for the clinical benefit of synchronously inhibiting both the primary driver mutation and the emerged putative resistance-driver alteration in the setting of acquired resistance to EGFR-TKIs [36,37], such a combinatorial targeted approach may also be successful at baseline to tackle inherently resistant co-mutated tumors. In this regard, the molecular techniques utilized in clinical routine, especially at diagnosis (PCR panels, targeted NGS, FISH, IHC and others), cover only a specified number of driver genes resulting in restricted knowledge of the elements regulating response and resistance to TKIs. Additionally, lung cancer is a very complex and heterogeneous disease characterized by spatially and temporally diverse combinations of mutations. Thus, the optimal implementation of combinatorial targeted therapy strategies for NSCLC in the future will require wider information on the genetic and epigenetic events that can lead to TKI-resistance and that could represent additional targets and predictive biomarkers. Relatedly, recent reports provide definite support to the application of extensive molecular profiling of NSCLC and other solid cancers. This approach may detect multiple molecular alterations that may coexist within individual tumors and may potentially represent actionable targets for combinatorial therapies in a significant number of patients [10,36,37,284,285].

Current updated international guidelines recommend that NSCLC patients with verified or probable adenocarcinoma histology or those with mixed histology including an adenocarcinoma component, younger NSCLC patients, and patients without a history of smoking, should be tested for *EGFR-*mutations, *ALK-*fusions and *ROS1-*fusions to identify candidates to first-line therapy with specific TKIs [49,50]. As we discussed above, the response to these drugs is variable and there is mounting evidence for the occurrence of co-mutations in other cancer-driver genes that may either cause initial resistance or reduce the time to progression to first-line TKI-treatment. These co-existing molecular alterations are becoming more effectively identifiable with the continuous technological progress of sensitive and specific comprehensive methods of massively parallel sequencing. Although these procedures are still technically and economically challenging for routine practice in pathology laboratories, the benefit obtained by multiplexed genetic sequencing panels is becoming widely recognized and makes them preferable to multiple single-gene tests for identifying mechanisms of TKI-resistance, alternative targets, and combined or sequential treatment options beyond *EGFR*, *ALK*, and *ROS1* [49,50].

Overall, these considerations suggest that, in addition to the three “must-be-tested” *EGFR, ALK* and *ROS1* genes (currently together with the assessment of PD-L1 status by IHC), testing of NSCLC should be expanded to include all classes of genomic alterations (base substitutions, indels, CNVs, and rearrangements) and detect other potential molecular biomarkers that could aid in more effectively predicting the response to first-line TKIs alone or combined with other drugs. For these reasons, the current updated guidelines also state that, given the growing knowledge on cancer-drivers involved in the development, progression, and therapy-resistance of NSCLC as well as the increase of molecularly targeted drugs, it is appropriate to include *BRAF, KRAS, MET, ERBB2, RET, NTRK* as part of larger multiplexed NGS testing panels performed either initially or when routine *EGFR, ALK,* and *ROS1* testing are negative [49,50]. Thus, it is expectable that with further understanding of the mechanisms of intrinsic and acquired drug-resistance, future guidelines will include recommendations for larger gene panels capable of impacting decisions regarding the first and following lines of targeted treatment for *EGFR*M+ NSCLC patients. The investigation of new TKIs of fourth generation, such as mutant-selective allosteric inhibitors capable of simultaneously inhibiting sensitizing *EGFR*-mutations, T790M, and C797S (and similar resistant mutations), as well as targeted drug combinations capable of overcoming resistance to the currently used EGFR-TKIs and improving the outcome of specific subgroups of *EGFR*M+ NSCLC patients is ongoing [286,287]. Consequently, the implementation of multiplexed molecular diagnostics is likely to become essential for better therapeutic strategies and prediction.

However, a significant challenge for the future development of effective multiplexed predictive tests and combinatorial treatment regimens is represented by genomic tumor heterogeneity and the multiplicity as well as unpredictability of TKI-resistance mechanisms. Targeting single genetic alterations, such as *EGFR*-mutants does not seem sufficient to ensure long-lasting or even curative tumor regressions. Thus, the mechanisms of intrinsic TKI-resistance, ideally, should be identified before treatment and the latter should be tailored according to the results of pre-treatment tests. Likewise, it is important to define the impact on the response and resistance to EGFR-TKIs of other recurrent genetic alterations downstream EGFR that have frequently been detected in LAC and that are attractive potential therapeutic targets. Such mutations affect the chromatin-modifying genes *SETD2, ARID1A*, and *SMARCA4*, the RNA-splicing genes *RBM10* and *U2AF1*, members of the oxidative stress-related Keap1-Nrf2 pathway, as well as the *MYC* proto-oncogene and genes of cell cycle regulation and WNT/β-catenin pathway [3,7,10,12,288]. Equally, further knowledge on the consequences of DNA damage/repair and genomic/chromosomal instability in NSCLC is urgently warranted. Limiting the occurrence of these processes that can result in significant SNVs and CNVs of many genes may at least in part prevent the occurrence of genomic heterogeneity, drug resistance, and tumor progression [11].

Furthermore, discovering common convergent diagnostic and therapeutic themes related to EGFR-TKI resistance is needed for tackling the challenge of tumor heterogeneity. In this respect, signaling players downstream EGFR appear as promising factors for counteracting TKI-resistance. One of these could be the TF NF-κB, which is activated in response to EGFR-TKIs, drives survival of EGFR-dependent cancer cells, and whose genetic or pharmacologic inhibition can potentiate erlotinib-induced apoptosis in NSCLC models [289,290]. Accordingly, increased expression of the NF-κB inhibitor IκB was predictive for positive response to EGFR-TKIs in *EGFR*M+ NSCLC patients [290]. Thus, the analysis of NF-κB/IκB expression was proposed as companion predictive marker for a potential combinatorial therapy pharmacologically targeting NF-κB in *EGFR*M+ NSCLC [290]. Another attractive element for tackling TKI-resistance downstream EGFR is AKT, as it has recently been shown that activation of the AKT pathway is a convergent trait in *EGFR*M+ NSCLCs with acquired resistance to EGFR-TKIs caused by different underlying mechanisms. Correspondingly, combined treatment with AKT- and EGFR-inhibitors synergistically inhibits the growth of preclinical models of *EGFR*M+ NSCLC resistant to erlotinib, gefitinib or osimertinib [291]. Importantly, phosphorylated AKT (pAKT) was detected by IHC not only in 60% of examined samples from NSCLC patients after progression on EGFR-TKIs by different resistance mechanisms, but also in 11% of baseline samples, suggesting the pre-existence of pAKT-positive, intrinsically resistant clones. Indeed, the pAKT-positive baseline cases displayed significantly worse PFS and OS to first-line EGFR-TKI therapy than pAKT-negative cases [291]. These data suggest that: (1) the analysis of pAKT levels at baseline may have clinical utility as a molecular predictor of response and resistance to EGFR-TKIs; and (2) AKT may be an attractive target for tackling intrinsic and acquired TKI-resistance. Similarly, recent preclinical studies have suggested that NSCLC cells made osimertinib-resistant through different mechanisms maintain their growth in part by aberrant EGFR-independent activation of the MAPK pathway downstream EGFR and can regain drug-sensitivity by combining osimertinib with a MEK-inhibitor [292,293]. Thus, co-targeting EGFR and downstream MAPK and AKT pathways might turn out to be an effective strategy to overcome resistance to EGFR-TKIs of different generations in the future.

In conclusion, there is a plethora of recognized, interchangeably dominating mechanisms that can cause intrinsic and/or acquired resistance to EGFR-TKIs, though many more are expected to be discovered, not least when osimertinib becomes the SOC first-line EGFR-TKI. For many patients with advanced *EGFR*M+ NSCLC the estimated median OS is reaching three years, thanks to the subsequent or combined employment of EGFR-TKIs and chemotherapy or immunotherapy. Yet, despite the five EGFR-TKIs (gefitinib, erlotinib, afatinib, dacomitinib, and osimertinib) currently available for the treatment of *EGFR*M+ NSCLC, the ideal sequence for administering these drugs remains to be established [294]. By the same token, there are several first-line options available for treating *EGFR*M+ NSCLC (i.e., 1G, 2G, and 3G TKIs, TKI+antiangiogenic agent and TKI+chemotherapy) after the report of the remarkable PFS benefit and immature OS data for osimertinib vs. 1G EGFR-TKIs and of the ARCHER phase III study displaying the superior PFS and OS benefit of the 2G TKI dacomitinib vs. 1G TKI [24,295,296]. Thus, elucidating how primary and acquired TKI-resistance may develop during these different therapeutic approaches is also important for individually choosing the optimal treatment for each patient. Therefore, (re)biopsies of tumor tissue and plasma cfDNA at baseline and progression represent an invaluable tool for detecting the individual resistance mechanisms in each patient and guiding further treatment of this very heterogenous disease. Especially, the study of signaling pathways downstream EGFR is expected to unveil new converging elements that can aid in predicting and treating intrinsic and acquired resistance to EGFR-TKIs.

## Figures and Tables

**Table 1:** *EGFR*-mutations associated with reduced response or primary resistance to EGFR-TKIs in NSCLC.

Somatic Mutation (Amino Acid Position)	Exon	Effect on EGFR-TKIs	Other Features	References
G719X S768I L861Q	18 20 21	Reduced response to 1G TKIs in pts. & preclinical models. Sensitive to afatinib. Osimertinib less effective in pts. or cell lines with these mutants than in those with classic EGFR-mutants, regardless of presence of T790M co-mutation.	Significantly less sensitive than L858R & exon 19dels but do show some response to 1G TKIs. Can co-occur together or with sensitizing mutations, especially L858R. The rare variant L861P reported co-existing with L858R in pts. not responding to 1G EGFR-TKIs.	[54,76,81,83,87,89,90,92,94]
L747P	19	Intrinsic resistance to EGFR-TKIs of all three generations	Very rare, resistance mechanism unclear. The variant L747S occasionally reported both as secondary TKI-resistant mutant in the setting of acquired TKI-resistance and as de novo mutation in cases with co-existing L858R not responding to 1G EGFR-TKIs.	[54,57,58,86,99,101]
Exon 19 insertions	19	Unclear (very rare, require further investigations)	Some epidemiological evidence for lower TKI-sensitivity than common EGFR-mutations.	[51,97,98]
Exon 20 insertions	20	Poor response to 1G/2G TKIs; in vitro appear responsive to osimertinib & single cases were reported sensitive to osimertinib.	A763_Y764insFQEA is an exception, as structurally resembles L858R & is sensitive to TKIs. In preclinical models, exon 20ins responded to cetuximab + afatinib or osimertinib. Cases responding to afatinib + cetuximab have been reported. Promising results in vitro and in vivo from new selective TKIs targeting EGFR and ERBB2 exon 20 insertions, such as poziotinib, TAS6417, and TAK-788. Heat shock protein 90 inhibitors also potentially active against NSCLC cells with EGFR exon 20ins.	[83,86,87,103,104,106,108,110,112]
T790M	20	Resistant to 1G/2G TKIs, sensitive to 3G TKIs.	Present as de novo mutation, either alone or with a common sensitizing mutation such as L858R. Amplification of T790M-positive EGFR may provide further TKI-resistance. High relative abundance of T790M predicts poor response to 1G/2G TKIs but may predict better response to 3G TKIs.	[51,54,57,61,62]
Germline T790M	20	Resistant to 1G/2G TKIs, sensitive to 3G TKIs.	Predominantly in females, non-smokers with a secondary somatic EGFR-mutation.	[115]
Germline V843I	21	Resistant to 1G/2G TKIs, possibly sensitive to 3G TKIs.	As T790M sterically hinders TKI-binding to EGFR.	[113,114,115]
